# Systematic review: comparative effectiveness of adjunctive devices in patients with ST-segment elevation myocardial infarction undergoing percutaneous coronary intervention of native vessels

**DOI:** 10.1186/1471-2261-11-74

**Published:** 2011-12-20

**Authors:** Diana M Sobieraj, C Michael White, Jeffrey Kluger, Vanita Tongbram, Jennifer Colby, Wendy T Chen, Sagar S Makanji, Soyon Lee, Ajibade Ashaye, Craig I Coleman

**Affiliations:** 1 University of Connecticut/Hartford Hospital Evidence-based Practice Center, Hartford, CT, USA

## Abstract

**Background:**

During percutaneous coronary intervention (PCI), dislodgement of atherothrombotic material from coronary lesions can result in distal embolization, and may lead to increased major adverse cardiovascular events (MACE) and mortality. We sought to systematically review the comparative effectiveness of adjunctive devices to remove thrombi or protect against distal embolization in patients with ST-segment elevation myocardial infarction (STEMI) undergoing PCI of native vessels.

**Methods:**

We conducted a systematic literature search of Medline, the Cochrane Database, and Web of Science (January 1996-March 2011), http://www.clinicaltrials.gov, abstracts from major cardiology meetings, TCTMD, and CardioSource Plus. Two investigators independently screened citations and extracted data from randomized controlled trials (RCTs) that compared the use of adjunctive devices plus PCI to PCI alone, evaluated patients with STEMI, enrolled a population with 95% of target lesion(s) in native vessels, and reported data on at least one pre-specified outcome. Quality was graded as good, fair or poor and the strength of evidence was rated as high, moderate, low or insufficient. Disagreement was resolved through consensus.

**Results:**

37 trials met inclusion criteria. At the maximal duration of follow-up, catheter aspiration devices plus PCI significantly decreased the risk of MACE by 27% compared to PCI alone. Catheter aspiration devices also significantly increased the achievement of ST-segment resolution by 49%, myocardial blush grade of 3 (MBG-3) by 39%, and thrombolysis in myocardial infarction (TIMI) 3 flow by 8%, while reducing the risk of distal embolization by 44%, no reflow by 48% and coronary dissection by 70% versus standard PCI alone. In a majority of trials, the use of catheter aspiration devices increased procedural time upon qualitative assessment.

Distal filter embolic protection devices significantly increased the risk of target revascularization by 39% although the use of mechanical thrombectomy or embolic protection devices did not significantly impact other final health outcomes. Distal balloon or any embolic protection device increased the achievement of MBG-3 by 61% and 20% and TIMI3 flow by 11% and 6% but did not significantly impact other intermediate outcomes versus control. Upon qualitative analysis, all device categories, with exception of catheter aspiration devices, appear to significantly prolong procedure time compared to PCI alone while none appear to significantly impact ejection fraction. Many of the final health outcome and adverse event evaluations were underpowered and the safety of devices overall is unclear due to insufficient amounts of data.

**Conclusions:**

In patients with STEMI, for most devices, few RCTs evaluated final health outcomes over a long period of follow-up. Due to insufficient data, the safety of these devices is unclear.

## Background

Over 650,000 deaths were attributed to coronary heart disease (CHD) in the United States in 2003 [1]. Coronary stents and adjunctive pharmacologic agents have improved the effect of percutaneous coronary intervention (PCI), establishing near normal antegrade blood flow in the vast majority of patients [1-4]. However, dislodgement of atherothrombotic material from coronary lesions during PCI can result in distal embolization, termed the "no-reflow phenomenon, in 12 to 39 percent of patients" [1,2].

Patients with no-reflow may have larger infarcts, more significant left ventricular systolic dysfunction, and an increased risk of major adverse cardiovascular events (MACE) or death. Numerous adjunctive devices have been developed to remove thrombi or protect against distal embolization during PCI [5]. These devices utilize different technologies and can be broadly classified as catheter aspiration, mechanical thrombectomy, or embolic protection devices (i.e., distal embolic balloon or filter protection devices or proximal embolic balloon protection devices) [6]. Distal embolic protection devices are recommended to be used in patients undergoing PCI of saphenous vein grafts due to previously demonstrated ability to reduce MACE [1,2]. However, use of embolic protection devices in STEMI has been less well supported mainly because of underpowered clinical trials that evaluated intermediate markers [2]. More recently, larger randomized controlled trials (RCTs) of patients with STEMI have evaluated MACE as an end point and followed patients beyond hospital discharge (typically 3 to 12 months) but have given conflicting results [7-17]. The Agency for Healthcare Research and Quality commissioned this report to systematically review the comparative effectiveness of adjunctive devices to remove thrombi or protect against distal embolization in patients with acute coronary syndromes (ACS) undergoing PCI of native vessels.

## Methods

We developed and followed a standard protocol for all steps of this review, which underwent review by a panel of experts in the field as well as the public. The peer-reviewed final report details the methodology including the analytic framework, literature search strategy, and analysis plan as well as evidence tables and is available at http://www.effectivehealthcare.ahrq.gov. The authors have no conflicts of interest.

We refined key questions in collaboration with a panel of technical experts which included cardiologists, internists, and representatives from managed-care organizations. The following key questions in patients with STEMI who are undergoing PCI of native vessels were defined:

1. What are the comparative effects of adjunctive devices from different classes on intermediate [e.g. ST-segment resolution (STSR), myocardial blush grade (MBG), thrombolysis in myocardial infarction (TIMI) 3 flow, ejection fraction, no reflow and distal embolization)] and final health outcomes [mortality, MACE, and health-related quality-of-life (HRQoL)]?

2. How do the rate and type of harms (e.g. coronary dissection, coronary perforation, and prolonged procedure time) differ between device types when compared to PCI alone?

The final report of this comparative effectiveness review, available at http://www.effectivehealthcare.ahrq.gov, includes information and results of analyses specific to patients with non-ST-segment myocardial infarction (NSTEMI), unstable angina (UA), or mixed ACS (STEMI, NSTEMI, and/or UA), results from observational trials with over 500 patients, and information on results in various subpopulations.

### Data Sources

We conducted a computerized literature search of Medline, Cochrane Central Register of Controlled Trials, Cochrane Database of Systematic Reviews, and Web of Science databases from January 1996- March 2010, without language restrictions. We restricted the search to 1996 and later to reflect contemporary practice. The complete search strategy is included in Additional File 1. Additionally, in an attempt to locate unpublished studies and increase the sensitivity of our search, we reviewed references from identified studies and systematic reviews. We searched for and reviewed abstracts from major cardiology meetings (American Heart Association, American College of Cardiology, European Society of Cardiology), Transcatheter Cardiovascular Therapeutics (TCT) Conference of the Cardiovascular Research Foundation and the TCTMD http://www.tctmd.com, CardioSource Plus http://www.cardiosource.com, and http://ClinicalTrials.govhttp://www.clinicaltrials.gov web sites. We updated the literature search in March 2011 during the peer review period using the same search strategy.

### Study Selection

Two independent reviewers assessed studies for inclusion in a parallel manner using *a priori *criteria. RCTs of any size were eligible for inclusion if they: 1) compared the use of adjunctive devices plus PCI to PCI alone, 2) included patients with STEMI, 3) had ≤ 5% of the study population receiving PCI of saphenous veins, and (4) reported data on at least one pre-specified intermediate outcome, final health outcome, or harm. Given the known benefit of distal embolic protection devices in patients undergoing PCI of a saphenous vein graft, this review was restricted to a population with lesions primarily in native vessels.

### Data Extraction

Two reviewers used a standardized data extraction tool to independently extract study data. Data extracted from each study included interventions, study design, inclusion and exclusion criteria, methodological quality criteria, study population, baseline patient characteristics, use of concurrent standard medical therapies, and pre-specified benefits and harms.

### Assessment of Study Quality and Strength of Evidence

Two reviewers independently assessed the validity and strength of evidence using recommendations in the Methods Guide for Effectiveness and Comparative Effectiveness Reviews [18]. We assessed each study for the following individual criteria: randomization technique, comparable study groups at baseline, detailed description of study outcomes, blinding of outcome assessors, intent-to-treat analysis, description of participant withdrawals (percent follow-up), and potential conflict of interest. Studies were then given an overall quality score of good, fair, or poor.

We used a modified version of the Grading of Recommendations Assessment, Development and Evaluation (GRADE) system to assess the strength of evidence for each outcome of interest. Four required domains were evaluated- risk of bias, consistency, directness, and precision. We classified the strength of evidence for each outcome as high, moderate, low, or insufficient.

### Data Synthesis and Analysis

We qualitatively examined data from all identified studies. Six device classes were considered and for each outcome, we conducted separate analyses of studies that compare each device class with control or two device classes to each other. Device classes included catheter aspiration, mechanical thrombectomy, distal filter embolic protection, distal balloon embolic protection, proximal balloon embolic protection, and embolic protection devices combined (distal or proximal, balloon or filter). We conducted meta-analyses when two or more RCTs that were adequate for data pooling were available for any outcome. For dichotomous outcomes, weighted averages are reported as relative risks (RR) and risk differences (RD) with associated 95 percent confidence intervals using a DerSimonian and Laird random-effects model [19]. We used automatic 'zero cell' correction for studies with no events for a particular outcome occurring in one group. We excluded studies with no events occurring in the treatment and control groups. We addressed statistical heterogeneity using the I^2 ^statistic (with a value > 50% deemed to be representative of significant heterogeneity) and we used Egger's weighted regression statistic to assess for the presence of publication bias [20]. We used StatsDirect statistical software, version 2.7.8 (StatsDirect Ltd., Cheshire, England) with a p-value of < 0.05 considered statistically significant.

We defined attainment of optimal myocardial reperfusion as a MBG-3 or TIMI-3 blood flow (or a MBG or TIMI blood flow of at least two in studies not reporting the other endpoint) and ST-segment resolution as 70 percent resolution in peak ST-segments (or at least 50 percent resolution in studies not reporting the other endpoint). We used results for ST-segment resolution at or close to 60 minutes post-PCI and never exceeding 90 minutes post-PCI. For final health outcomes, we defined the base-case analysis using the maximum duration of follow-up for which a final health outcome was reported.

To assess the effect of heterogeneity (both clinical and methodological) on the conclusions of our meta-analysis, we conducted subgroup and sensitivity analyses based on study quality (limited to good quality trials) and duration of follow-up on the efficacy of adjunctive devices. Data at different follow-up times (in-hospital, ≥ 30 days but < 180 days, ≥ 180 days but < 365 days, and ≥ 365 days) were pooled in separate subgroup analyses.

### Role of the Funding Source

The University of Connecticut/Hartford Hospital Evidence-based Practice Center prepared this systematic review, with funding from the Agency of Healthcare Research and Quality. The funding source formulated draft research questions and provided the copyright release for this manuscript, but did not participate in the literature search, data collection or analysis, or interpretation of the results.

## Results

**Figure 1:**
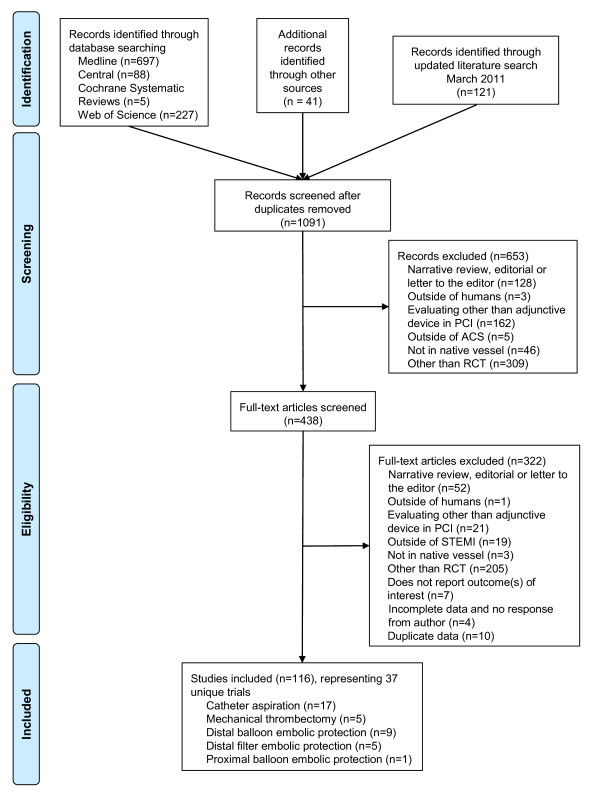
**Preferred Reporting Items for Systematic Reviews and Meta-Analyses**. Diagram Abbreviations: n = number; PCI = percutaneous coronary intervention; PRISMA = preferred reporting items for systematic reviews and meta-analyses; RCT = randomized controlled trial; STEMI = ST segment elevation myocardial infarction.

We screened 1,091 abstracts and 438 full text articles (Figure 1). Specific to the STEMI population, we included 37 unique RCTs [7-17,21-47].

### Evidence of Benefit with Adjunctive Devices in patients with STEMI

All RCTs compared an adjunctive device plus PCI to standard PCI alone. Compared to standard PCI, 17 trials evaluated catheter aspiration devices [8-12,15-17,21-30] (n = 3355; 11 good, 3 fair and 3 poor quality trials), 5 trials evaluated mechanical thrombectomy devices [7,31-34] (n = 1374; 5 good quality), 9 trials evaluated distal balloon embolic protection devices [13,35-42] (n = 1479; 8 good and 1 fair quality), 5 trials evaluated distal filter embolic protection devices [43-47] (n = 962; 4 good and 1 fair quality), and trial evaluated proximal balloon embolic protection devices [14] (n = 284, good quality).

The baseline characteristics of included trials are presented in Additional File 2, Table S1. The mean age of patients enrolled in the 37 trials ranged from 55 to 69 years presenting within 6 to 48 hours of symptom onset. Twenty-one of the 34 trials included patients presenting within 12 hours of symptom onset. Males constituted at least half of the patients in the trials, ranging from 55 to 95 percent of the total population. The mean ischemic time reported in the 37 trials ranged from 120 to 510 minutes. The percent of patients presenting with TIMI 0/1 at baseline ranged from 55 to 100 percent. Of the 37 trials, 24 trials included patients with no prior fibrinolysis before the index PCI. Five trials included patients with prior fibrinolysis as well as primary PCI and eight trials did not report whether patients who received prior fibrinolysis were included.

### Final Health Outcomes

#### Maximum Duration of Follow-Up Analyses

The use of catheter aspiration devices plus PCI significantly decreased the risk of MACE compared to PCI alone by 27% and showed trends (upper or lower extent of the 95% confidence intervals within 0.05 units of the line of unity) towards reducing mortality, myocardial infarction, and target revascularization versus PCI alone (Table 1). Distal filter embolic protection devices significantly increased the risk of target revascularization by 61% compared to PCI alone. Mechanical thrombectomy, distal balloon embolic protection, proximal balloon embolic protection, and the combined group of embolic protection devices plus PCI failed to significantly impact any final health outcome versus PCI alone (Additional file 3 to 25). All of the adjunctive devices non-significantly increased the risk of stroke versus PCI alone. Limiting studies to those of good methodological quality did not alter any of these results. No trials evaluated the impact of adjunctive devices on HRQoL.

**Table 1:** Effect of adjunctive devices on final health outcomes

Outcome	N trials/N pooled	WMF (M)	RR (95% CI)	I^2^	SOE*	N trials/N pooled	WMF (M)	RR (95% CI)	I^2^
	***All eligible trials***					***Limited to good quality trials***			

**Catheter Aspiration**									
Mortality	11/10	7.92	0.69 (0.47 to 1.02)	0%	Low	10/10	8.08	0.70 (0.47 to 1.03)	0%
Myocardial infarction	10/10	8.80	0.61 (0.36 to 1.04)	0%	Low	10/10	8.80	0.61 (0.36 to 1.04)	0%
Stroke	5/4	0.79	3.18 (0.73 to 13.88)	0%	Insufficient	5/4	0.79	3.18 (0.73 to 13.88)	0%
Target revascularization	9/9	9.48	0.79 (0.61 to 1.02)	0%	Low	9/9	9.48	0.79 (0.61 to 1.02)	0%
MACE	11/11	12.43	0.73 (0.61 to 0.88)	0%	High	11/11	12.43	0.73 (0.61 to 0.88)	0%
HRQoL	0	---	---	---	Insufficient	0	---	---	---

**Mechanical Thrombectomy**									
Mortality	5/4	7.80	1.19 (0.51 to 2.76)	54.9	Insufficient	5/4	7.80	1.19 (0.51 to 2.76)	54.9
Myocardial infarction	5/3	8.98	0.71 (0.27 to 1.85)	0%	Insufficient	5/3	8.98	0.71 (0.27 to 1.85)	0%
Stroke	5/4	5.79	2.42 (0.75 to 7.78)	0%	Insufficient	5/4	5.79	2.42 (0.75 to 7.78)	0%
Target revascularization	5/3	6.22	0.87 (0.36 to 2.10)	39.2%	Insufficient	5/3	6.22	0.87 (0.36 to 2.10)	39.2%
MACE	4/3	6.22	1.23 (0.50 to 3.01)	79.9%	Insufficient	4/3	6.22	1.23 (0.50 to 3.01)	79.9%
HRQoL	0	---	---	---	Insufficient	0	---	---	---

**Distal Filter Embolic Protection**									
Mortality	5/5	10.84	0.97 (0.54 to 1.75)	0%	Insufficient	4/4	11.49	0.97 (0.53 to 1.79)	0%
Myocardial infarction	5/4	11.22	0.72 (0.15 to 3.34)	39.8%	Insufficient	4/3	11.93	0.56 (0.06 to 5.02)	60%
Stroke	1/0	1	1.51 (0.30 to 7.52)^†^	NA	Insufficient	1/0	1	1.51 (0.30 to 7.52)^†^	NA
Target revascularization	3/2	13.36	1.61 (1.03 to 2.54)	NA	Low	3/2	13.36	1.61 (1.03 to 2.54)	NA
MACE	5/5	10.84	1.34 (0.97 to 1.86)	0%	Moderate	4/4	11.49	1.36 (0.98 to 1.89)	0%
HRQoL	0	---	---	---	Insufficient	0	---	---	---

**Distal Balloon Embolic Protection**									
Mortality	4/4	6	0.82 (0.45 to 1.51)	2.5%	Insufficient	4/4	6	0.82 (0.45 to 1.51)	2.5%
Myocardial infarction	5/5	6	0.67 (0.29 to 1.57)	0%	Insufficient	5/5	6	0.67 (0.29 to 1.57)	0%
Stroke	1	6	0.48 (0.10 to 2.22)^†^	NA	Insufficient	1/0	6	0.48 (0.10 to 2.22)^†^	NA
Target revascularization	5/5	6	0.93 (0.61 to 1.42)	0%	Insufficient	5/5	6	0.93 (0.61 to 1.42)	0%
MACE	6/5	6	0.87 (0.64 to 1.19)	0%	Insufficient	6/5	6	0.87 (0.64 to 1.19)	0%
HRQoL	0	---	---	---	Insufficient	0	---	---	---

**Proximal Balloon Embolic Protection**									
Mortality	1/0	6	0.51 (0.11 to 2.33)^†^	NA	Insufficient	1/0	6	0.51 (0.11 to 2.33)^†^	NA
Myocardial infarction	1/0	6	1.01 (0.24 to 4.33)^†^	NA	Insufficient	1/0	6	1.01 (0.24 to 4.33)^†^	NA
Stroke	1/0	6	0.20 (0 to 1.93)^†^	NA	Insufficient	1/0	6	0.20 (0 to 1.93)^†^	NA
Target revascularization	1/0	6	0.71 (0.29 to 1.75)^†^	NA	Insufficient	1/0	6	0.71 (0.29 to 1.75)^†^	NA
MACE	1/0	6	0.74 (0.36 to 1.54)^†^	NA	Insufficient	1/0	6	0.74 (0.36 to 1.54)^†^	NA
HRQoL	0	---	---	---	Insufficient	0	---	---	---

**Embolic Protection Devices**									
Mortality	10/10	8.11	0.87 (0.58 to 1.30)	0%	Insufficient	9/9	8.31	0.87 (0.57 to 1.31)	0%
Myocardial infarction	11/10	8.08	0.83 (0.45 to 1.53)	0%	Insufficient	10/9	8.27	0.83 (0.45 to 1.55)	0%
Stroke	3/3	3.74	0.68 (0.22 to 2.11)	0%	Insufficient	3/3	3.74	0.68 (0.22 to 2.11)	0%
Target revascularization	9/8	8.60	1.11 (0.80 to 1.52)	10%	Insufficient	9/8	8.60	1.11 (0.80 to 1.52)	10%
MACE	12/11	7.97	1.04 (0.84 to 1.29)	0%	Moderate	11/10	8.15	1.03 (0.82 to 1.29)	4%
HRQoL	0	---	---	---	Insufficient	0	---	---	---

Abbreviations: CI = confidence interval; HRQoL = health-related quality of life; m = months; MACE = major adverse cardiovascular events; N = number; NA = not applicable; RR = relative risk; SOE = strength of evidence; WMF = weighted mean follow-up

* Strength of evidence is rated for the primary base analyses only. Subgroup analyses were not rated with strength of evidence; include limiting the analysis to trials of good methodological quality.

†Result is based on a single trial

#### Time Specified Analyses

When we evaluated final health outcomes over several different time periods, we found several significant findings (Table 2). Catheter aspiration devices plus PCI significantly reduced the risk of mortality at 365 days while target revascularization and MACE were significantly reduced at 180 days versus PCI alone. Mechanical aspiration devices plus PCI reduced target revascularization at 180 days and MACE at 365 days versus PCI alone. Distal filter embolic protection devices plus PCI paradoxically significantly increased the risk of target revascularization and MACE at 365 days versus PCI alone. Based on a single RCT, distal balloon embolic protection devices plus PCI significantly reduced the risk of in hospital and 90 day stroke versus PCI alone.

**Table 2:** Effect of adjunctive devices on final health outcomes using different follow-up

Outcome	WMF (M)	≤ 30d RR (95% CI)	In-hospital RR (95% CI)	30d RR (95% CI)	180d RR (05% CI)	365d RR (95% CI)
**Catheter Aspiration**						
Mortality	0.79	0.65 (0.39 to 1.10)	0.81 (0.23 to 2.86)	0.61 (0.35 to 1.07)	0.89 (0.31 to 2.51)	0.62 (0.39 to 0.98)
Myocardial infarction	0.77	0.55 (0.24 to 1.25)	0.32 (0.03 to 3.06)	0.60 (0.25 to 1.45)	0.70 (0.24 to 1.99)	0.51 (0.26 to 1.00)
Stroke	0.79	3.18 (0.73 to 13.88)	4.94 (0.52 to infinity)	2.77 (0.51 to 14.98)	---*	---†
Target revascularization	0.70	0.85 (0.53 to 1.38)	1.35 (0.26 to 6.94)	0.82 (0.50 to 1.35)	0.62 (0.40 to 0.96)	0.87 (0.63 to 1.19)
MACE	0.79	0.80 (0.57 to 1.12)	0.97 (0.36 to 2.58)	0.79 (0.56 to 1.13)	0.66 (0.47 to 0.94)	0.61 (0.26 to 1.41)
HRQoL	---	---	---	---	---	---

**Mechanical Thrombectomy**						
Mortality	1	1.25 (0.47 to 3.32)	1.00 (0.24 to 4.16)‡	1.25 (0.47 to 3.32)	1.35 (0.53 to 3.44)	0.50 (0.21 to 1.17)
Myocardial infarction	1	0.63 (0.21 to 1.96)	1.00 (0.11 to 9.41)‡	0.63 (0.21 to 1.96)	0.57 (0.17 to 1.92)	0.66 (0.13 to 3.29)
Stroke	1	1.89 (0.55 to 6.48)	---*	1.89 (0.55 to 6.48)	2.05 (0.27 to 15.78)	1.99 (0.26 to 15.14)
Target revascularization	1	1.62 (0.21 to 12.55)	---*	1.62 (0.21 to 12.55)	0.55 (0.33 to 0.92)	0.68 (0.41 to 1.13)
MACE	1	1.28 (0.37 to 4.38)	---†	1.28 (0.37 to 4.38)	0.71 (0.41 to 1.20)	0.66 (0.44 to 0.97)
HRQoL	---	---	---	---	---	---

**Distal Filter Embolic Protection**						
Mortality	1	1.02 (0.50 to 2.08)	---†	1.02 (0.50 to 2.08)	1.25 (0.38 to 4.16)‡	0.87 (0.43 to 1.78)‡
Myocardial infarction	1	0.73 (0.12 to 4.44)	---†	0.73 (0.12 to 4.44)	0.09 (0 to 0.74)*	2.35 (0.61 to 8.90)‡
Stroke	1	1.51 (0.30 to 7.52)‡	---†	1.51 (0.30 to 7.52)‡	---†	---†
Target revascularization	1	3.02 (0.61 to 14.84)	---†	3.02 (0.70 to 13.01)‡	1.00 (0.35 to 2.82)‡	1.78 (1.09 to 2.93)‡
MACE	1	1.29 (0.77 to 2.15)	---†	1.29 (0.77 to 2.15)	1.10 (0.68 to 1.78)	1.48 (1.03 to 2.15)‡
HRQoL	---	---	---	---	---	---

**Distal Balloon Embolic Protection**						
Mortality	1	0.64 (0.30 to 1.39)	0.69 (0.24 to 2.03)‡	0.64 (0.30 to 1.39)	0.86 (0.48 to 1.57)	---†
Myocardial infarction	1	0.85 (0.32 to 2.23)	0.32 (0.00 to 3.71)‡	0.85 (0.32 to 2.23)	0.67 (0.29 to 1.57)	---†
Stroke	1	0.11 (0 to 0.94)‡	---†	0.11 (0 to 0.94)‡	0.48 (0.10 to 2.22)‡	---†
Target revascularization	1	1.38 (0.55 to 3.50)	0.32 (0.00 to 3.71)‡	1.38 (0.55 to 3.50)	0.93 (0.61 to 1.42)	---†
MACE	1	0.74 (0.44 to 1.23)	---*	0.74 (0.44 to 1.23)	0.87 (0.64 to 1.19)	---†
HRQoL	---	---	---	---	---	---

**Proximal Balloon Embolic Protection**						
Mortality	1	1.01 (0.14 to 7.10)‡	---†	1.01 (0.18 to 5.69)‡	0.51 (0.11 to 2.33)‡	---†
Myocardial infarction	1	0.68 (0.11 to 3.99)‡	---†	0.68 (0.14 to 3.34)‡	1.01 (0.24 to 4.33)‡	---†
Stroke	1	0.34 (0.01 to 8.23)‡	---†	0.34 (0 to 3.87)‡	0.20 (0.00 to 1.93)‡	---†
Target revascularization	1	0.51 (0.13 to 1.99)‡	---†	0.51 (0.14 to 1.81)‡	0.71 (0.29 to 1.75)‡	---†
MACE	1	0.61 (0.23 to 1.63)‡	---†	0.61 (0.23 to 1.57)‡	0.74 (0.36 to 1.54)‡	---†
HRQoL	---	---	---	---	---	---

**Embolic Protection Devices**						
Mortality	1	0.84 (0.50 to 1.39)	0.69 (0.24 to 2.03)‡	0.84 (0.50 to 1.39)	0.87 (0.52 to 1.46)	0.87 (0.43 to 1.78)‡
Myocardial infarction	1	0.83 (0.41 to 1.69)	0.32 (0.00 to 3.71)‡	0.83 (0.41 to 1.69)	0.65 (0.31 to 1.33)	2.35 (0.61 to 8.90)‡
Stroke	1	0.56 (0.11 to 2.84)	---†	0.56 (0.11 to 2.84)	0.39 (0.09 to 1.71)	---†
Target revascularization	1	1.24 (0.62 to 2.48)	0.32 (0.00 to 3.71)‡	1.24 (0.62 to 2.48)	0.90 (0.63 to 1.30)	1.78 (1.09 to 2.93)‡
MACE	1	0.92 (0.66 to 1.30)	---*	0.92 (0.66 to 1.30)	0.91 (0.71 to 1.16)	1.48 (1.03 to 2.15)‡
HRQoL	---	---	---	---	---	---

Abbreviations: d = day; CI = confidence interval; HRQoL = health-related quality of life; m = months; MACE = major adverse cardiovascular events; N = number; = relative risk; WMF = weighted mean follow-up

* Risk could not be calculated because no events occurred in the trial evaluating this outcome

† Risk could not be calculated because no trials evaluated this outcome

‡ Result is based on a single trial

### Intermediate Health Outcomes

The use of catheter aspiration devices plus PCI significantly increased the achievement of STSR by 51%, MBG-3 by 61% and TIMI-3 flow by 8% while they significantly decreased the risk of no reflow by 52% and distal embolization by 56% versus PCI alone (Table 3 and Additional file 26 to 49). The use of either distal balloon embolic protection device or the combined group of embolic protection devices plus PCI significantly increased achievement of MBG-3 (39% and 20%, respectively) and TIMI-3 flow (11% and 6%, respectively) versus PCI alone. Embolic protection devices showed a trend towards improvement in attaining STSR, however, neither device category significantly impacted STSR, distal embolization, and no reflow versus PCI alone. Mechanical thrombectomy devices, distal filter embolic protection and proximal balloon embolic protection devices failed to significantly impact STSR, MBG-3, TIMI-3, no reflow and distal embolization, although no trials evaluated proximal embolic balloon protection devices on the risk of no reflow versus PCI alone. All three device categories demonstrated trends toward improvement in attaining STSR and MBG while proximal balloon embolic protection devices showed trends toward improvement in MBG-3 versus PCI alone. However, the proximal balloon embolic protection device data were based on a single trial. Limiting studies to those of good methodological quality did not alter any of these results.

**Table 3:** Effect of adjunctive devices on intermediate health outcomes and harms

Outcome	N trials/N pooled	Relative Risk (95% CI)	I^2^	Strength of Evidence*	N trials/ N pooled	Relative Risk (95% CI)	I^2^
**Intermediate Outcomes**							

	***All eligible trials***				***Limited to good quality trials***		

**Catheter Aspiration**							
ST-segment resolution	15/15	1.51 (1.32 to 1.73)	64.2%	Moderate	10/10	1.39 (1.21 to 1.61)	60.4%
MBG-3	13/13	1.61 (1.41 to 1.84)	55.4%	Moderate	9/9	1.75 (1.44 to 2.14)	69.2%
TIMI-3	13/13	1.08 (1.04 to 1.12)	11.5%	Moderate	10/10	1.07 (1.04 to 1.11)	0%
Distal embolization	10/10	0.56 (0.39 to 0.79)	43.4%	High	8/8	0.48 (0.34 to 0.66)	33.7%
No reflow	8/8	0.52 (0.35 to 0.76)	15.7%	High	6/6	0.45 (0.27 to 0.75)	22.3%

**Mechanical Thrombectomy**							
ST-segment resolution	5/5	1.16 (0.99 to 1.36)	75.1%	Low	5/5	1.16 (0.99 to 1.36)	75.1%
MBG-3	4/4	1.07 (0.80 to 1.43)	76.5%	Low	4/4	1.07 (0.80 to 1.43)	76.5%
TIMI-3	4/4	0.98 (0.92 to 1.04)	67.5%	Moderate	4/4	0.98 (0.92 to 1.04)	67.5%
Distal embolization	3/3	0.44 (0.17 to 1.12)	41.6%	Moderate	3/3	0.44 (0.17 to 1.12)	41.6%
No reflow	3/3	0.50 (0.17 to 1.48)	41.7%	Insufficient	3/3	0.50 (0.17 to 1.48)	41.7%

**Distal Filter Embolic Protection**							
ST-segment resolution	5/5	1.05 (0.97 to 1.15)	0%	Moderate	4/4	1.05 (0.96 to 1.14)	0%
MBG-3	2/2	0.97 (0.81 to 1.15)	NA	Moderate	2/2	0.97 (0.81 to 1.15)	NA
TIMI-3	5/5	1.00 (0.90 to 1.11)	69.6%	Low	4/4	1.02 (0.90 to 1.15)	70.2%
Distal embolization	1/0	0.63 (0.22 to 1.82)†	NA	Insufficient	1/0	0.63 (0.22 to 1.82)†	NA
No reflow	2/2	0.59 (0.14 to 2.51)	NA	Insufficient	1/0	1.00 (0.18 to 5.55)†	NA

**Distal Balloon Embolic Protection**							
ST-segment resolution	4/4	1.08 (0.91 to 1.29)	41.2%	Moderate	4/4	1.08 (0.91 to 1.29)	41.2%
MBG-3	6/6	1.39 (1.15 to 1.69)	43.5%	High	6/6	1.39 (1.15 to 1.69)	43.5%
TIMI-3	9/8	1.11 (1.03 to 1.19)	60.4%	Low	8/7	1.09 (1.01 to 1.17)	59.7%
Distal embolization	4/4	1.10 (0.67 to 1.81)	5.8%	Moderate	4/4	1.10 (0.67 to 1.81)	5.8%
No reflow	4/4	0.51 (0.19 to 1.33)	0%	Insufficient	4/4	0.51 (0.19 to 1.33)	0%

**Proximal Balloon Embolic Protection**							
ST-segment resolution	1/0	1.11 (0.97 to 1.28)†	NA	Insufficient	1/0	1.11 (0.97 to 1.28)†	NA
MBG-3	1/0	0.98 (0.88 to 1.10)†	NA	Insufficient	1/0	0.98 (0.88 to 1.10)†	NA
TIMI-3	1/0	1.06 (0.98 to 1.15)†	NA	Insufficient	1/0	1.06 (0.98 to 1.16)†	NA
Distal embolization	1/0	0.71 (0.37 to 1.35)†	NA	Insufficient	1/0	0.71 (0.38 to 1.33)†	NA
No reflow	1/0	---‡	---‡	Insufficient	1/0	---‡	---‡

**Embolic Protection Devices**							
ST-segment resolution	10/10	1.06 (1.00 to 1.13)	0%	Low	10/10	1.06 (1.00 to 1.13)	0%
MBG-3	9/9	1.20 (1.02 to 1.40)	68.2%	Moderate	9/9	1.20 (1.02 to 1.40)	68.2%
TIMI-3	15/14	1.06 (1.01 to 1.12)	58.3%	Low	15/14	1.06 (1.01 to 1.12)	55.4%
Distal embolization	6/6	0.91 (0.64 to 1.30)	0.2%	Moderate	6/6	0.91 (0.64 to 1.30)	0.2%
No reflow	6/6	0.53 (0.24 to 1.18)	0%	Insufficient	5/5	0.58 (0.25 to 1.37)	0%

**Harms**							

**Catheter Aspiration**							
Coronary dissection	5/5	0.30 (0.12 to 0.75)	0%	High	5/5	0.30 (0.12 to 0.75)	0%
Coronary perforation	1/0	---‡	---‡	Insufficient	1/0	---‡	---‡

**Mechanical Thrombectomy**							
Coronary dissection	1/0	1.51 (0.57 to 4.01)†	NA	Insufficient	1/0	1.51 (0.57 to 4.01)†	NA
Coronary perforation	2/2	1.04 (0.15 to 7.04)	NA	Insufficient	2/2	1.04 (0.15 to 7.04)	NA

**Distal Filter Embolic Protection**							
Coronary dissection	1/0	---‡	---‡	Insufficient	1/0	---‡	---‡
Coronary perforation	1/0	---‡	---‡	Insufficient	1/0	---‡	---‡

**Distal Balloon Embolic Protection**							
Coronary dissection	1/0	---‡	---‡	Insufficient	1/0	---‡	---‡
Coronary perforation	1/0	5.11 (0.53 to infinity)†	NA	Insufficient	1/0	5.11 (0.53 to infinity) †	NA

**Proximal Balloon Embolic Protection**							
Coronary dissection	0/0	---	---	Insufficient	0/0	---	---
Coronary perforation	0/0	---	---	Insufficient	0/0	---	---

**Embolic Protection Devices**							
Coronary dissection	2/0	---‡	---‡	Insufficient	2/0	---‡	---‡
Coronary perforation	1/0	5.11 (0.53 to infinity)†	NA	Insufficient	1/0	5.11 (0.53 to infinity) †	NA

Abbreviations: MBG = myocardial blush grade; N = number; NA = not applicable; TIMI = thrombolysis in myocardial infarction

* Strength of evidence is rated for the primary base analyses only. Subgroup analyses were not rated with strength of evidence; include limiting the analysis to trials of good methodological quality.

† Result is based on a single trial

‡ Risk could not be calculated because no events occurred

We qualitatively evaluated ejection fraction for each device category versus standard PCI (Table 4). Based on the majority of trials within each device category, none of the adjunctive devices categories appear to significantly impact ejection fraction versus standard PCI. However, data were inconsistently reported across trials and the time period in which ejection fraction was measured varied from in-hospital to 6 months post-PCI, preventing rational pooling of results.

**Table 4:** Ejection fraction and procedure time*

Study, Year	Group	n	Time EF Measured	Mean EF (SD)	P-value	n	Mean Procedure Time	P-value
		***Ejection Fraction ***				***Procedure Time***		

**Catheter Aspiration**								

Dudek, 2010	Diver CE Control	--- ---	---	--- ---	---	--- ---	--- ---	---

Liistro, 2009	Export Thrombectomy Catheter Control	55 56	180d	55 (6) 49 (8)	< 0.0001	55 56	75.7 (30.0) 75.9 (38.7)	0.90

Lipiecki, 2009	Export Catheter Control	20 24	7d	48 (12) 45 (11)	0.4	--- ---	--- ---	---

Moura, 2009	TAC Control	--- ---	---	--- ---	---	--- ---	--- ---	---

Sardella, 2009	Export Medtronic	38 37	3-5d	46.3 (8.6) 44.3 (9.5)	0.30	--- ---	--- ---	---
	(EM) Control	36 36	90d	49.0 (9.3) 46.7 (10.6)	0.3			

Wita, 2009	Diver CE Control	19 23	7d	50.1 (8.4) 46.5 (7.9)		19 23	39.5 (10.1) 32.3 (18.6)	0.14

Chao, 2008	Export Aspiration Catheter Control	37 37	28d	56 (10) 57 (10)	0.51	37 37	49 (18)^†^ 53 (23)^†^	0.54

Chevalier, 2008	Export Aspiration Catheter Control	--- ---	---	--- ---	---	120 129	36.7 (18.0) 34.5 (21.5)	0.08

Ciszewski, 2008	Rescue/Diver Control	32 31	5-8d	46.7 (11.0) 42.5 (10.0)	0.16	--- ---	--- ---	---

Ikari, 2008	TVAC Control	103 113	180d	57.1 (12.5) 56.7 (12.3)	0.77	178 180	87.0 (32.4) 93.6 (78.6)	0.16

Svilaas, 2008	6F Export Aspiration Catheter Control	--- ---	---	--- ---	---	535 536	28 (14-42)‡ 26 (12-40)‡	0.92

DeLuca, 2006	Diver CE Control	38 38	Post-PCI	37.29 (9.97) 36.67 (3.03)	> 0.05	--- ---	--- ---	---
		35 36	180d	42.97 (9.97) 41.28 (3.37)	> 0.05			

Kaltoft, 2006	Rescue Catheter Control	108 107	30d	51 (43-57)† 53 (47-58)†	0.13	108 107	39 (29-48)‡ 29 (23-38)‡	< 0.0001

Lee, 2006	Export Aspiration Catheter Control	--- ---	---	--- ---	---	--- ---	--- ---	---

Silva-Orrego, 2006	Pronto Extraction Catheter Control	--- ---	---	--- ---	---	74 74	57 (19) 54 (21)	0.36

Burzotta, 2005	Diver CE Control	25 25	1d	50.36 (8.76) 45.75 (7.49)	< 0.05	50 49	81 (43) 72 (34)	0.41
		25 25	7d	53.34 (10.99) 48.09 (9.4)	< 0.05			
		25 25	180d	53.28 (10.04) 47.72 (8.28)	< 0.05			

Noel, 2005	Export Control	--- ---	---	--- ---	---	--- ---	--- ---	---

Dudek, 2004	Rescue Control	35 32	In-hospital	56.5 (9.1) 52.8 (12.8)	> 0.05	--- ---	--- ---	---
		35 32	90d	60.3 (9.2) 55.3 (14.7)	> 0.05			

**Mechanical Thrombectomy**								

Migliorini, 2010	AngioJet Rheolytic Thrombectomy Control	--- ---	---	--- ---	---	256 245	59.5 (44.7-70)‡ 46 (35-60)‡	< 0.001

Ali, 2006	AngioJet Catheter Control	197 205	14-28d	51.3 (11.53) 52.3 (10.89)	0.38	240 240	75.4 (30.9) 59.2 (26.8)	< 0.001

Lefèvre, 2005	X-Sizer Catheter Control	--- ---	---	--- ---	---	100 101	54 (28) 45 (25)	0.009

Antoniucci, 2004	AngioJet Control	--- ---	---	--- ---	---	--- ---	--- ---	---

Napodano, 2003	X-Sizer Catheter Control	46 46	In-hospital	51.0 (7.7) 48.7 (10.9)	0.29	--- ---	--- ---	---

		46 46	30d	51.9 (7.9) 49.9 (8.9)	0.26			

**Distal Filter Embolic Protection Devices**								

Ito, 2010	Filtrap Control	--- ---	---	---	---	--- ---	--- ---	---

Kelbæk, 2008	FilterWire-EZ or SpiderX Control	--- ---	---	--- ---	---	--- ---	--- ---	---

Cura, 2007	SpideRX Control	70 70	2-3d	47.4 (9.9) 45.3 (7.3)	0.29	70 70	52 (43-70) ‡ 43.5 (30-54) ‡	< 0.001

Guetta, 2007	FilterWire EZ Control	51 49	Post-PCI	47 (---) 44 (---)	0.56	--- ---	--- ---	---

Lefèvre, 2004	AngioGuardXP Control	--- ---	---	--- ---	---	--- ---	--- ---	---

**Distal Balloon Embolic Protection Devices**								

Duan, 2010	PercuSurge Guardwire Plus	46 50	90d	51.6 (3.6) 49.3 (5.3)	< 0.05	--- ---	--- ---	---
	Control	46 50	180d	53.0 (3.7) 50.8 (5.2)	< 0.05			

Pan, 2010	PercuSurge Guardwire Control	--- ---	---	---	---	--- ---	--- ---	---

Tahk, 2008	PercuSurge GuardWire Control	48 47	180d	58.1 (11.4) 54.6 (10.3)	0.24	--- ---	--- ---	---

Hahn, 2007	GuardWire Control	19 20	3d	50 (9) 49 (13)	0.60	--- ---	--- ---	---
		15 14	180d	48 (16) 50 (9)	0.74			

Matsuo, 2007	GuardWire Distal Protection Control	80 74	Post-PCI	46.1 (9.5) 55.4 (13.9)	0.99	80 74	75.8 (30) 53 (25)	< 0.01
		80 74	180d	61.9 (---) 62.7 (---)	0.36			

Muramatsu, 2007	GuardWire Plus System Control	173 168	Post-PCI	54.0 (---) 53.8 (---)	0.90	173 168	29.7 (18.3)§ 29.5 (18.2)§	0.91
		133 123	30d	55.3 (---) 55.4 (---)	> 0.05			
		108 117	180d	57.1 (---) 57.1 (---)	> 0.05			

Zhou, 2007	PercuSurge GuardWire Control	--- ---	---	--- ---	---	--- ---	--- ---	---

Okamura, 2005	PercuSurge GuardWire Control	--- ---	D/c (mean 22 ± 4 d)	47 (9) 48 (8)	0.89	8 8	--- ---	---

Stone, 2005	GuardWire Plus Control	--- ---	---	--- ---	---	252 249	53 (42-69)‡ 39 (29-51)‡	< 0.001

**Proximal Balloon Embolic Protection Devices**								

Haeck, 2009	Proxis Control	96 110	4-6m	50 (11) 50 (12)	0.46	141 143	45 (36-58)‡ 31 (25-40)‡	< 0.01

*Strength of evidence ratings for ejection fraction and prolonged procedure time, respectively, are: Catheter aspiration device moderate: high; Mechanical Thrombectomy devices: moderate, high; Distal filter embolic protection devices: low, insufficient; Distal balloon embolic protection devices: moderate, low; proximal balloon embolic protection devices: insufficient, moderate; embolic protection devices combined: moderate, moderate

^† ^Lab to TIMI-3

‡ Median (interquartile range)

§ operation time

--- not reported

Abbreviations: d = days; d/c = discharge; EF = ejection fraction; n = number of participants included in the analysis of ejection fraction; PCI = percutaneous coronary intervention, SD = standard deviation; TAC = Thrombectomy Aspiration Catheter; TVAC = Transvascular aspiration catheter

### Harms

The use of catheter aspiration devices plus PCI significantly decreased the risk of coronary dissection by 70% compared to PCI alone and no events occurred in the one trial evaluating coronary perforation (Table 3 and Additional file 50 to 54). Mechanical thrombectomy, distal filter embolic protection, distal balloon embolic protection, and the combined group of embolic protection devices plus PCI failed to significantly impact or show trends towards impacting these harms versus PCI alone. However, analyses were generally limited to a small number of trials and in some cases no events occurred so the risk of harms could not be calculated. Limiting studies to those of good methodological quality did not alter any of these results. No trials evaluated these harms for proximal balloon embolic protection devices.

Catheter aspiration devices plus PCI does not appear to prolong procedure time since a majority of trials (8 of 9 trials) evaluating this outcome found no significant differences versus PCI alone (Table 4). Mechanical thrombectomy (3 of 3 trials), distal balloon embolic protection (2 of 3 trials), distal filter embolic protection (1 of 1 trial) proximal balloon embolic protection devices (1 of 1 trial) and embolic protection devices (4 of 5 trials) plus PCI appear to prolong procedure time versus standard PCI.

## Discussion

Determining the balance of benefits to harms is difficult because many of the final health outcome and adverse event evaluations were underpowered and the safety of devices overall is unclear due to insufficient amounts of data. We do not know for certain whether the non-significant increases or decreases observed were due to a real effect or to chance.

In the catheter aspiration trials, the risk of MACE and coronary dissection were significantly lower in the overall analyses and the good quality trial analyses. However, the risk of mortality, myocardial infarction, and target revascularization were not significantly impacted (although there was a trend in the right direction) and a non-significant increase in the risk of stroke was found. However, STSR, MBG-3, TIMI-3, no reflow, and distal embolization were favorably impacted by catheter aspiration devices compared to standard PCI. As such, more research is needed to truly determine the balance of benefits to harms but use of catheter aspiration devices looks promising.

Mechanical thrombectomy device use did not result in any significant differences in the final health outcomes or coronary dissection and coronary perforation in the overall analyses and analyses limited to good quality trials. However, these devices significantly increased the time needed to conduct the PCI procedure in three trials. While the risk of myocardial infarction, target revascularization, mortality and MACE were not significantly different versus control, these findings may be misleading since many of the trials evaluating this procedure versus control had a shorter duration of follow-up. When we evaluated mortality and MACE in studies of 365 days or longer, there was no significant difference in mortality risk although there was a significant reduction in MACE, based on the results of a single trial. Unlike with catheter aspiration devices, there are no significant beneficial effects on intermediate health outcomes and while most are in the right direction of effect, the chance of achieving near normal (TIMI-3) blood flow was not significantly different versus control. As such, more research is needed to truly determine the balance of benefits to harms with mechanical thrombectomy devices.

The use of embolic protection devices was based on a limited number of studies and one significant finding (the positive impact of distal filter devices on target revascularization) on final health outcomes was seen in overall analyses and those limited to good quality trials. It was difficult to assess the impact on final health outcomes and intermediate outcomes for these devices. Distal balloon devices significantly increased the chance of achieving a MBG-3 or near normal (TIMI-3) blood flow but did not significantly impact the achievement of STSR, prevention of no reflow, or the risk of distal embolization. Distal filter devices did not significantly impact STSR, distal embolization, no reflow, attainment of near normal (TIMI-3) blood flow, or MBG. There was a paucity of trials available to evaluate adverse events with any of the embolic protection devices. The only significant finding was an increased time to perform a PCI procedure for all three types of embolic protection devices individually and when evaluated together versus control. As such, the balance of benefits to harms cannot be determined for these device classes.

The use of thrombus removal and embolic protection devices hold promise in the adjunctive treatment of patients with STEMI undergoing primary PCI. However, to truly discern the role of these devices in contemporary practice, a number of important research questions need to be answered. In our analysis, we found that for many endpoints, non-significant increases or decreases were seen versus control, even when we evaluated compound endpoints, used the maximum duration of follow-up, and combined three different types of embolic protection devices together. All of these were strategies to enhance power to detect differences between groups but by and large, did not provide adequate power. Ultimately, the impact of using these devices on long-term final health outcomes versus control needs to be determined.

Previous systematic reviews have attempted to address this topic. However, only four of 11 which were identified through our systematic literature search comprehensively included devices from all three device categories including catheter aspiration devices, mechanical thrombectomy, and embolic protection [48-51]. Albeit comprehensive in the devices evaluated, only the meta-analysis by Bavry and colleagues, published in 2008, attempted to evaluate a duration of follow-up beyond 30 days for final health outcomes, including stroke, MACE, and its components [50]. However, this analysis did not include safety outcomes and since then, additional RCTs have been published, which are included in our analyses.

Based on these research gaps we propose the following avenues for future research. We believe that additional multicenter, randomized, placebo-controlled trials should be conducted to determine the impact of adjunctive clot removal or embolic protection devices on final health outcomes using a long term follow-up versus PCI alone. At least two such trials are currently ongoing, Thrombus Aspiration in STEMI in Scandinavia (TASTE) and a Trial of Routine Aspiration Thrombectomy with PCI versus PCI alone in Patients with STEMI Undergoing Primary PCI (TOTAL) [52,53]. Both trials plan to have a longer duration of follow-up, with the TASTE trial following patients for 10 years and TOTAL up to 1 year. To truly determine comparative effectiveness, the devices found to have the best balance of benefits to harms compared with standard PCI should be directly compared in a multicenter, randomized, active controlled trial to determine the impact of adjunctive clot removal or embolic protection devices on final health outcomes using a long term follow-up. Such trials should have international representation of interventional cardiologists and include both tertiary academic medical centers and large community based hospitals. Conducting these additional clinical trials would facilitate the conduction of mixed treatment meta-analyses or individual patient data meta-analyses to estimate the comparative effectiveness of different device classes.

## Conclusion

In patients with STEMI, for most devices, few RCTs evaluated final health outcomes over a long period of follow-up. Due to insufficient data, the safety of these devices is unclear.

## Abbreviations

ACS: acute coronary syndrome; CHD: coronary heart disease; HRQoL: health-related quality of life; MACE: major adverse cardiovascular event; MBG: myocardial blush grade; NSTEMI: non-ST-segment elevation myocardial infarction; PCI: percutaneous coronary intervention; RCT: randomized controlled trial; RD: risk difference; RR: relative risk; STEMI: ST-segment elevation myocardial infarction; STSR: ST-segment resolution; TCT: transcatheter cardiovascular therapeutics; TIMI: time in myocardial infarction; UA: unstable angina.

## Competing interests

The authors declare that they have no competing interests.

## Authors' contributions

DMS, CMW, JK and CIC contributed to the conception of design, acquisition, analysis and interpretation of data, and drafted the manuscript. VT, JC, WTC, SSM, SL, and AA contributed to the acquisition and analysis of data and drafted the manuscript. All authors read and approved the final manuscript.

## Pre-publication history

The pre-publication history for this paper can be accessed here:

http://www.biomedcentral.com/1471-2261/11/74/prepub

## Supplementary Material

Additional file 1 **Literature search strategy**. This file contains the literature search strategy used for this review. Click here for file

Additional file 2 **Table S1**. Baseline study characteristics. This file contains an additional table with included trial baseline characteristics. Click here for file

Additional file 3 **Impact of catheter aspiration devices versus control on mortality using the maximal duration of followup in patients with ST-segment elevation myocardial infarction**. Figure of the Impact of catheter aspiration devices versus control on mortality using the maximal duration of followup in patients with ST-segment elevation myocardial infarction. The squares represent individual point estimates. The size of the square represents the weight given to each study in the meta-analysis. Horizontal lines through each square represent 95 percent confidence intervals. The diamond represents the combined results. The solid vertical line extending from 1 is the null value. Click here for file

Additional file 4 **Impact of mechanical thrombectomy devices versus control on mortality using the maximal duration of followup in patients with ST-segment elevation myocardial infarction**. Figure of the Impact of mechanical thrombectomy devices versus control on mortality using the maximal duration of followup in patients with ST-segment elevation myocardial infarction. The squares represent individual point estimates. The size of the square represents the weight given to each study in the meta-analysis. Horizontal lines through each square represent 95 percent confidence intervals. The diamond represents the combined results. The solid vertical line extending from 1 is the null value. Click here for file

Additional file 5 **Impact of distal filter embolic protection devices versus control on mortality using the maximal duration of followup in patients with ST- segment elevation myocardial infarction**. Figure of the Impact of distal filter embolic protection devices versus control on mortality using the maximal duration of followup in patients with ST- segment elevation myocardial infarction. The squares represent individual point estimates. The size of the square represents the weight given to each study in the meta-analysis. Horizontal lines through each square represent 95 percent confidence intervals. The diamond represents the combined results. The solid vertical line extending from 1 is the null value. Click here for file

Additional file 6 **Impact of distal balloon embolic protection devices versus control on mortality using the maximal duration of followup versus control in patients with ST-segment elevation myocardial infarction**. Figure of the Impact of distal balloon embolic protection devices versus control on mortality using the maximal duration of followup versus control in patients with ST-segment elevation myocardial infarction. The squares represent individual point estimates. The size of the square represents the weight given to each study in the meta-analysis. Horizontal lines through each square represent 95 percent confidence intervals. The diamond represents the combined results. The solid vertical line extending from 1 is the null value. Click here for file

Additional file 7 **Impact of embolic protection devices combined versus control on mortality using the maximal duration of followup in patients with ST- segment elevation myocardial infarction**. Figure of the Impact of embolic protection devices combined versus control on mortality using the maximal duration of followup in patients with ST-segment elevation myocardial infarction. The squares represent individual point estimates. The size of the square represents the weight given to each study in the meta-analysis. Horizontal lines through each square represent 95 percent confidence intervals. The diamond represents the combined results. The solid vertical line extending from 1 is the null value. Click here for file

Additional file 8 **Impact of catheter aspiration devices versus control on myocardial infarction using the maximal duration of followup in patients with ST-segment elevation myocardial infarction**. Figure of the Impact of catheter aspiration devices versus control on myocardial infarction using the maximal duration of followup in patients with ST-segment elevation myocardial infarction. The squares represent individual point estimates. The size of the square represents the weight given to each study in the meta-analysis. Horizontal lines through each square represent 95 percent confidence intervals. The diamond represents the combined results. The solid vertical line extending from 1 is the null value. Click here for file

Additional file 9 **Impact of mechanical thrombectomy devices versus control on myocardial infarction using the maximal duration of followup in patients with ST-segment elevation myocardial infarction**. Figure of the Impact of mechanical thrombectomy devices versus control on myocardial infarction using the maximal duration of followup in patients with ST-segment elevation myocardial infarction. The squares represent individual point estimates. The size of the square represents the weight given to each study in the meta-analysis. Horizontal lines through each square represent 95 percent confidence intervals. The diamond represents the combined results. The solid vertical line extending from 1 is the null value. Click here for file

Additional file 10 **Impact of distal filter embolic protection devices versus control on myocardial infarction using the maximal duration of followup in patients with ST-segment elevation myocardial infarction**. Figure of the Impact of distal filter embolic protection devices versus control on myocardial infarction using the maximal duration of followup in patients with ST-segment elevation myocardial infarction. The squares represent individual point estimates. The size of the square represents the weight given to each study in the meta-analysis. Horizontal lines through each square represent 95 percent confidence intervals. The diamond represents the combined results. The solid vertical line extending from 1 is the null value. Click here for file

Additional file 11 **Impact of distal balloon embolic protection devices versus control on myocardial infarction using the maximal duration of followup in patients with ST-segment elevation myocardial infarction**. Figure of the Impact of distal balloon embolic protection devices versus control on myocardial infarction using the maximal duration of followup in patients with ST-segment elevation myocardial infarction. The squares represent individual point estimates. The size of the square represents the weight given to each study in the meta-analysis. Horizontal lines through each square represent 95 percent confidence intervals. The diamond represents the combined results. The solid vertical line extending from 1 is the null value. Click here for file

Additional file 12 **Impact of embolic protection devices combined versus control on myocardial infarction using the maximal duration of followup in patients with ST-segment elevation myocardial infarction**. Figure of the Impact of embolic protection devices combined versus control on myocardial infarction using the maximal duration of followup in patients with ST-segment elevation myocardial infarction. The squares represent individual point estimates. The size of the square represents the weight given to each study in the meta-analysis. Horizontal lines through each square represent 95 percent confidence intervals. The diamond represents the combined results. The solid vertical line extending from 1 is the null value. Click here for file

Additional file 13 **Impact of catheter aspiration devices versus control on stroke using the maximal duration of followup in patients with ST-segment elevation myocardial infarction**. Figure of the Impact of catheter aspiration devices versus control on stroke using the maximal duration of followup in patients with ST-segment elevation myocardial infarction. The squares represent individual point estimates. The size of the square represents the weight given to each study in the meta-analysis. Horizontal lines through each square represent 95 percent confidence intervals. The diamond represents the combined results. The solid vertical line extending from 1 is the null value. Click here for file

Additional file 14 **Impact of mechanical thrombectomy devices versus control on occurrence of stroke using the maximal duration of followup in patients with ST-segment elevation myocardial infarction**. Figure of the Impact of mechanical thrombectomy devices versus control on occurrence of stroke using the maximal duration of followup in patients with ST-segment elevation myocardial infarction. The squares represent individual point estimates. The size of the square represents the weight given to each study in the meta-analysis. Horizontal lines through each square represent 95 percent confidence intervals. The diamond represents the combined results. The solid vertical line extending from 1 is the null value. Click here for file

Additional file 15 **Impact of embolic protection devices combined versus control on stroke using the maximal duration of followup in patients with ST-segment elevation myocardial infarction**. Figure of the Impact of embolic protection devices combined versus control on stroke using the maximal duration of followup in patients with ST-segment elevation myocardial infarction. The squares represent individual point estimates. The size of the square represents the weight given to each study in the meta-analysis. Horizontal lines through each square represent 95 percent confidence intervals. The diamond represents the combined results. The solid vertical line extending from 1 is the null value. Click here for file

Additional file 16 **Impact of catheter aspiration devices versus control on target revascularization using the maximal duration of followup in patients with ST-segment elevation myocardial infarction**. Figure of the Impact of catheter aspiration devices versus control on target revascularization using the maximal duration of followup in patients with ST-segment elevation myocardial infarction. The squares represent individual point estimates. The size of the square represents the weight given to each study in the meta-analysis. Horizontal lines through each square represent 95 percent confidence intervals. The diamond represents the combined results. The solid vertical line extending from 1 is the null value. Click here for file

Additional file 17 **Impact of mechanical thrombectomy devices versus control on target revascularization using the maximal duration of followup in patients with ST-segment elevation myocardial infarction**. Figure of the Impact of mechanical thrombectomy devices versus control on target revascularization using the maximal duration of followup in patients with ST-segment elevation myocardial infarction. The squares represent individual point estimates. The size of the square represents the weight given to each study in the meta-analysis. Horizontal lines through each square represent 95 percent confidence intervals. The diamond represents the combined results. The solid vertical line extending from 1 is the null value. Click here for file

Additional file 18 **Impact of distal filter embolic protection devices versus control on target revascularization using the maximal duration of followup in patients with ST-segment elevation myocardial infarction**. Figure of the Impact of distal filter embolic protection devices versus control on target revascularization using the maximal duration of followup in patients with ST-segment elevation myocardial infarction. The squares represent individual point estimates. The size of the square represents the weight given to each study in the meta-analysis. Horizontal lines through each square represent 95 percent confidence intervals. The diamond represents the combined results. The solid vertical line extending from 1 is the null value. Click here for file

Additional file 19 **Impact of distal balloon embolic protection devices versus control on target revascularization using maximal duration of followup in patients with ST-segment elevation myocardial infarction**. Figure of the Impact of distal balloon embolic protection devices versus control on target revascularization using maximal duration of followup in patients with ST-segment elevation myocardial infarction. The squares represent individual point estimates. The size of the square represents the weight given to each study in the meta-analysis. Horizontal lines through each square represent 95 percent confidence intervals. The diamond represents the combined results. The solid vertical line extending from 1 is the null value. Click here for file

Additional file 20 **Impact of embolic protection devices combined versus control on target revascularization using the maximal duration of followup in patients with ST-segment elevation myocardial infarction**. Figure of the Impact of embolic protection devices combined versus control on target revascularization using the maximal duration of followup in patients with ST-segment elevation myocardial infarction. The squares represent individual point estimates. The size of the square represents the weight given to each study in the meta-analysis. Horizontal lines through each square represent 95 percent confidence intervals. The diamond represents the combined results. The solid vertical line extending from 1 is the null value. Click here for file

Additional file 21 **Impact of catheter aspiration devices versus control on MACE of maximal duration of followup in patients with ST-segment elevation myocardial infarction**. Figure of the Impact of catheter aspiration devices versus control on MACE of maximal duration of followup in patients with ST-segment elevation myocardial infarction. The squares represent individual point estimates. The size of the square represents the weight given to each study in the meta-analysis. Horizontal lines through each square represent 95 percent confidence intervals. The diamond represents the combined results. The solid vertical line extending from 1 is the null value. Click here for file

Additional file 22 **Impact of mechanical thrombectomy devices versus control on MACE using the maximal duration of followup in patients with ST-segment elevation myocardial infarction**. Figure of the Impact of mechanical thrombectomy devices versus control on MACE using the maximal duration of followup in patients with ST-segment elevation myocardial infarction. The squares represent individual point estimates. The size of the square represents the weight given to each study in the meta-analysis. Horizontal lines through each square represent 95 percent confidence intervals. The diamond represents the combined results. The solid vertical line extending from 1 is the null value. Click here for file

Additional file 23 **Impact of distal filter embolic protection devices versus control on MACE using the maximal duration of followup in patients with ST-segment elevation myocardial infarction**. Figure of the Impact of distal filter embolic protection devices versus control on MACE using the maximal duration of followup in patients with ST-segment elevation myocardial infarction. The squares represent individual point estimates. The size of the square represents the weight given to each study in the meta-analysis. Horizontal lines through each square represent 95 percent confidence intervals. The diamond represents the combined results. The solid vertical line extending from 1 is the null value. Click here for file

Additional file 24 **Impact of distal balloon embolic protection devices versus control on MACE using the maximal duration of followup in patients with ST-segment elevation myocardial infarction**. Figure of the Impact of distal balloon embolic protection devices versus control on MACE using the maximal duration of followup in patients with ST-segment elevation myocardial infarction. The squares represent individual point estimates. The size of the square represents the weight given to each study in the meta-analysis. Horizontal lines through each square represent 95 percent confidence intervals. The diamond represents the combined results. The solid vertical line extending from 1 is the null value. Click here for file

Additional file 25 **Impact of embolic protection devices combined versus control on MACE using the maximal duration of followup in patients with ST-segment elevation myocardial infarction**. Figure of the Impact of embolic protection devices combined versus control on MACE using the maximal duration of followup in patients with ST-segment elevation myocardial infarction. The squares represent individual point estimates. The size of the square represents the weight given to each study in the meta-analysis. Horizontal lines through each square represent 95 percent confidence intervals. The diamond represents the combined results. The solid vertical line extending from 1 is the null value. Click here for file

Additional file 26 **Impact of catheter aspiration devices versus control on ST-segment resolution in patients with ST-segment elevation myocardial infarction**. Figure of the Impact of catheter aspiration devices versus control on ST-segment resolution in patients with ST-segment elevation myocardial infarction. The squares represent individual point estimates. The size of the square represents the weight given to each study in the meta-analysis. Horizontal lines through each square represent 95 percent confidence intervals. The diamond represents the combined results. The solid vertical line extending from 1 is the null value. Click here for file

Additional file 27 **Impact of mechanical thrombectomy devices versus control on ST-segment resolution in patients with ST-segment elevation myocardial infarction Figure of the Impact of mechanical thrombectomy devices versus control on ST-segment resolution in patients with ST-segment elevation myocardial infarction**. The squares represent individual point estimates. The size of the square represents the weight given to each study in the meta-analysis. Horizontal lines through each square represent 95 percent confidence intervals. The diamond represents the combined results. The solid vertical line extending from 1 is the null value. Click here for file

Additional file 28 **Impact of distal filter embolic protection devices versus control on ST-segment resolution in patients with ST-segment elevation myocardial infarction**. Figure of the Impact of distal filter embolic protection devices versus control on ST-segment resolution in patients with ST-segment elevation myocardial infarction. The squares represent individual point estimates. The size of the square represents the weight given to each study in the meta-analysis. Horizontal lines through each square represent 95 percent confidence intervals. The diamond represents the combined results. The solid vertical line extending from 1 is the null value. Click here for file

Additional file 29 **Impact of distal balloon embolic protection devices versus control on ST-segment resolution in patients with ST-segment elevation myocardial infarction**. Figure of the Impact of distal balloon embolic protection devices versus control on ST-segment resolution in patients with ST-segment elevation myocardial infarction. The squares represent individual point estimates. The size of the square represents the weight given to each study in the meta-analysis. Horizontal lines through each square represent 95 percent confidence intervals. The diamond represents the combined results. The solid vertical line extending from 1 is the null value. Click here for file

Additional file 30 **Impact of embolic protection devices combined versus control on ST-segment resolution in patients with ST-segment elevation myocardial infarction**. Figure of the Impact of embolic protection devices combined versus control on ST-segment resolution in patients with ST-segment elevation myocardial infarction. The squares represent individual point estimates. The size of the square represents the weight given to each study in the meta-analysis. Horizontal lines through each square represent 95 percent confidence intervals. The diamond represents the combined results. The solid vertical line extending from 1 is the null value. Click here for file

Additional file 31 **Impact of catheter aspiration devices versus control on myocardial blush grade of 3 in patients with ST-segment elevation myocardial infarction**. Figure of the Impact of catheter aspiration devices versus control on myocardial blush grade of 3 in patients with ST-segment elevation myocardial infarction. The squares represent individual point estimates. The size of the square represents the weight given to each study in the meta-analysis. Horizontal lines through each square represent 95 percent confidence intervals. The diamond represents the combined results. The solid vertical line extending from 1 is the null value. Click here for file

Additional file 32 **Impact of mechanical thrombectomy devices versus control on myocardial blush grade of 3 in patients with ST-segment elevation myocardial infarction**. Figure of the Impact of mechanical thrombectomy devices versus control on myocardial blush grade of 3 in patients with ST-segment elevation myocardial infarction. The squares represent individual point estimates. The size of the square represents the weight given to each study in the meta-analysis. Horizontal lines through each square represent 95 percent confidence intervals. The diamond represents the combined results. The solid vertical line extending from 1 is the null value. Click here for file

Additional file 33 **Impact of distal filter embolic protection devices versus control on myocardial blush grade of 3 in patients with ST-segment elevation myocardial infarction**. Figure of the Impact of distal filter embolic protection devices versus control on myocardial blush grade of 3 in patients with ST-segment elevation myocardial infarction. The squares represent individual point estimates. The size of the square represents the weight given to each study in the meta-analysis. Horizontal lines through each square represent 95 percent confidence intervals. The diamond represents the combined results. The solid vertical line extending from 1 is the null value. Click here for file

Additional file 34 **Impact of distal balloon embolic protection devices versus control on myocardial blush grade of 3 in patients with ST-segment elevation myocardial infarction**. Figure of the Impact of distal balloon embolic protection devices versus control on myocardial blush grade of 3 in patients with ST-segment elevation myocardial infarction. The squares represent individual point estimates. The size of the square represents the weight given to each study in the meta-analysis. Horizontal lines through each square represent 95 percent confidence intervals. The diamond represents the combined results. The solid vertical line extending from 1 is the null value. Click here for file

Additional file 35 **Impact of embolic protection devices combined versus control on myocardial blush grade of 3 in patients with ST-segment elevation myocardial infarction**. Figure of the Impact of embolic protection devices combined versus control on myocardial blush grade of 3 in patients with ST-segment elevation myocardial infarction. The squares represent individual point estimates. The size of the square represents the weight given to each study in the meta-analysis. Horizontal lines through each square represent 95 percent confidence intervals. The diamond represents the combined results. The solid vertical line extending from 1 is the null value. Click here for file

Additional file 36 **Impact of catheter aspiration devices versus control on TIMI-3 blood flow in patients with ST-segment elevation myocardial infarction**. Figure of the Impact of catheter aspiration devices versus control on TIMI-3 blood flow in patients with ST-segment elevation myocardial infarction. The squares represent individual point estimates. The size of the square represents the weight given to each study in the meta-analysis. Horizontal lines through each square represent 95 percent confidence intervals. The diamond represents the combined results. The solid vertical line extending from 1 is the null value. Click here for file

Additional file 37 **Impact of mechanical thrombectomy devices versus control on TIMI- 3 blood flow in patients with ST-segment elevation myocardial infarction**. Figure of the Impact of mechanical thrombectomy devices versus control on TIMI- 3 blood flow in patients with ST-segment elevation myocardial infarction. The squares represent individual point estimates. The size of the square represents the weight given to each study in the meta-analysis. Horizontal lines through each square represent 95 percent confidence intervals. The diamond represents the combined results. The solid vertical line extending from 1 is the null value. Click here for file

Additional file 38 **Impact of distal filter embolic protection devices versus control on TIMI-3 blood flow in patients with ST-segment elevation myocardial infarction**. Figure of the Impact of distal filter embolic protection devices versus control on TIMI-3 blood flow in patients with ST-segment elevation myocardial infarction. The squares represent individual point estimates. The size of the square represents the weight given to each study in the meta-analysis. Horizontal lines through each square represent 95 percent confidence intervals. The diamond represents the combined results. The solid vertical line extending from 1 is the null value. Click here for file

Additional file 39 **Impact of distal balloon embolic protection devices versus control on TIMI- 3 blood flow in patients with ST-segment elevation myocardial infarction**. Figure of the Impact of distal balloon embolic protection devices versus control on TIMI- 3 blood flow in patients with ST-segment elevation myocardial infarction. The squares represent individual point estimates. The size of the square represents the weight given to each study in the meta-analysis. Horizontal lines through each square represent 95 percent confidence intervals. The diamond represents the combined results. The solid vertical line extending from 1 is the null value. Click here for file

Additional file 40 **Impact of embolic protection devices combined versus control on TIMI-3 blood flow in patients with ST-segment elevation myocardial infarction**. Figure of the Impact of embolic protection devices combined versus control on TIMI-3 blood flow in patients with ST-segment elevation myocardial infarction. The squares represent individual point estimates. The size of the square represents the weight given to each study in the meta-analysis. Horizontal lines through each square represent 95 percent confidence intervals. The diamond represents the combined results. The solid vertical line extending from 1 is the null value. Click here for file

Additional file 41 **Impact of catheter aspiration devices versus control on distal embolization in patients with ST-segment elevation myocardial infarction**. Figure of the Impact of catheter aspiration devices versus control on distal embolization in patients with ST-segment elevation myocardial infarction. The squares represent individual point estimates. The size of the square represents the weight given to each study in the meta-analysis. Horizontal lines through each square represent 95 percent confidence intervals. The diamond represents the combined results. The solid vertical line extending from 1 is the null value. Click here for file

Additional file 42 **Impact of mechanical thrombectomy devices versus control on distal embolization in patients with ST-segment elevation myocardial infarction**. Figure of the Impact of mechanical thrombectomy devices versus control on distal embolization in patients with ST-segment elevation myocardial infarction. The squares represent individual point estimates. The size of the square represents the weight given to each study in the meta-analysis. Horizontal lines through each square represent 95 percent confidence intervals. The diamond represents the combined results. The solid vertical line extending from 1 is the null value. Click here for file

Additional file 43 **Impact of distal balloon embolic protection devices versus control on distal embolization in patients with ST-segment elevation myocardial infarction**. Figure of the Impact of distal balloon embolic protection devices versus control on distal embolization in patients with ST-segment elevation myocardial infarction. The squares represent individual point estimates. The size of the square represents the weight given to each study in the meta-analysis. Horizontal lines through each square represent 95 percent confidence intervals. The diamond represents the combined results. The solid vertical line extending from 1 is the null value. Click here for file

Additional file 44 **Impact of embolic protection devices combined versus control on distal embolization in patients with ST-segment elevation myocardial infarction**. Figure of the Impact of embolic protection devices combined versus control on distal embolization in patients with ST-segment elevation myocardial infarction. The squares represent individual point estimates. The size of the square represents the weight given to each study in the meta-analysis. Horizontal lines through each square represent 95 percent confidence intervals. The diamond represents the combined results. The solid vertical line extending from 1 is the null value. Click here for file

Additional file 45 **Impact of catheter aspiration devices versus control on no reflow in patients with ST-segment elevation myocardial infarction**. Figure of the Impact of catheter aspiration devices versus control on no reflow in patients with ST-segment elevation myocardial infarction. The squares represent individual point estimates. The size of the square represents the weight given to each study in the meta-analysis. Horizontal lines through each square represent 95 percent confidence intervals. The diamond represents the combined results. The solid vertical line extending from 1 is the null value. Click here for file

Additional file 46 **Impact of mechanical thrombectomy devices versus control on no reflow in patients with ST-segment elevation myocardial infarction**. Figure of the Impact of mechanical thrombectomy devices versus control on no reflow in patients with ST-segment elevation myocardial infarction. The squares represent individual point estimates. The size of the square represents the weight given to each study in the meta-analysis. Horizontal lines through each square represent 95 percent confidence intervals. The diamond represents the combined results. The solid vertical line extending from 1 is the null value. Click here for file

Additional file 47 **Impact of distal filter embolic protection devices versus control on no reflow in patients with ST-segment elevation myocardial infarction**. Figure of the Impact of distal filter embolic protection devices versus control on no reflow in patients with ST-segment elevation myocardial infarction. The squares represent individual point estimates. The size of the square represents the weight given to each study in the meta-analysis. Horizontal lines through each square represent 95 percent confidence intervals. The diamond represents the combined results. The solid vertical line extending from 1 is the null value. Click here for file

Additional file 48 **Impact of distal balloon embolic protection devices versus control on no reflow in patients with ST-segment elevation myocardial infarction Figure of the Impact of distal balloon embolic protection devices versus control on no reflow in patients with ST-segment elevation myocardial infarction**. The squares represent individual point estimates. The size of the square represents the weight given to each study in the meta-analysis. Horizontal lines through each square represent 95 percent confidence intervals. The diamond represents the combined results. The solid vertical line extending from 1 is the null value. Click here for file

Additional file 49 **Impact of embolic protection devices combined versus control on no reflow in patients with ST-segment elevation myocardial infarction**. Figure of the Impact of embolic protection devices combined versus control on no reflow in patients with ST-segment elevation myocardial infarction. The squares represent individual point estimates. The size of the square represents the weight given to each study in the meta-analysis. Horizontal lines through each square represent 95 percent confidence intervals. The diamond represents the combined results. The solid vertical line extending from 1 is the null value. Click here for file

Additional file 50 **Impact of catheter aspiration devices on coronary dissection versus control in patients with ST-segment elevation myocardial infarction**. Figure of the Impact of catheter aspiration devices on coronary dissection versus control in patients with ST-segment elevation myocardial infarction. The squares represent individual point estimates. The size of the square represents the weight given to each study in the meta-analysis. Horizontal lines through each square represent 95 percent confidence intervals. The diamond represents the combined results. The solid vertical line extending from 1 is the null value. Click here for file

Additional file 51 **Impact of mechanical thrombectomy devices on coronary perforation versus control in patients with ST-segment elevation myocardial infarction**. Figure of the Impact of mechanical thrombectomy devices on coronary perforation versus control in patients with ST-segment elevation myocardial infarction. The squares represent individual point estimates. The size of the square represents the weight given to each study in the meta-analysis. Horizontal lines through each square represent 95 percent confidence intervals. The diamond represents the combined results. The solid vertical line extending from 1 is the null value. Click here for file

Additional file 52 **Impact of catheter aspiration devices on side branch occlusion versus control in patients with ST-segment elevation myocardial infarction**. Figure of the Impact of catheter aspiration devices on side branch occlusion versus control in patients with ST-segment elevation myocardial infarction. The squares represent individual point estimates. The size of the square represents the weight given to each study in the meta-analysis. Horizontal lines through each square represent 95 percent confidence intervals. The diamond represents the combined results. The solid vertical line extending from 1 is the null value. Click here for file

Additional file 53 **Impact of distal balloon embolic protection devices on side branch occlusion versus control in patients with ST-segment elevation myocardial infarction**. Figure of the Impact of distal balloon embolic protection devices on side branch occlusion versus control in patients with ST-segment elevation myocardial infarction. The squares represent individual point estimates. The size of the square represents the weight given to each study in the meta-analysis. Horizontal lines through each square represent 95 percent confidence intervals. The diamond represents the combined results. The solid vertical line extending from 1 is the null value. Click here for file

Additional file 54 **Impact of embolic protection devices combined on side branch occlusion versus control in patients with ST-segment elevation myocardial infarction**. Figure of the Impact of embolic protection devices combined on side branch occlusion versus control in patients with ST-segment elevation myocardial infarction. The squares represent individual point estimates. The size of the square represents the weight given to each study in the meta-analysis. Horizontal lines through each square represent 95 percent confidence intervals. The diamond represents the combined results. The solid vertical line extending from 1 is the null value. Click here for file
