# Supplementary material for: Systematic review: comparative effectiveness of adjunctive devices in patients with ST-segment elevation myocardial infarction undergoing percutaneous coronary intervention of native vessels
Source: BMC Cardiovasc Disord. 2011 Dec 20;11:74. doi: 10.1186/1471-2261-11-74 (PMC3313863; doi:10.1186/1471-2261-11-74)
Supplement: Additional file 1 — Literature search strategy. This file contains the literature search strategy used for this review. [file 1471-2261-11-74-S1.DOC]

**Additional file 1**

**Literature Search Strategy**

Search Strategy for MEDLINE, CENTRAL, CDSR (each in OVID starting in 1996), and Web of Science (limited to meeting abstracts only)

1. myocardial infarction.mp. or Myocardial Infarction/
2. acute myocardial infarction.mp.
3. AMI.mp.
4. MI.mp.
5. STEMI.mp.
6. ST-segment elevation.mp.
7. ACS.mp.
8. NSTEMI.mp.
9. acute coronary syndrome.mp. or Acute Coronary Syndrome/
10. ST-segment resolution.mp.
11. unstable angina.mp. or Angina, Unstable/
12. Q-wave.mp.
13. no-reflow.mp.
14. distal embolization.mp.
15. Angioplasty, Transluminal, Percutaneous Coronary/ or percutaneous coronary intervention.mp.
16. PCI.mp.
17. 1 or 2 or 3 or 4 or 5 or 6 or 7 or 8 or 9 or 10 or 11 or 12 or 13 or 14 or 15 or 16
18. thrombectomy.mp. or Thrombectomy/
19. embolic protection.mp.
20. distal protection.mp.
21. proximal protection.mp.
22. thrombus aspiration.mp.
23. aspiration catheter.mp.
24. rescue catheter.mp.
25. diver CE.mp.
26. Export catheter.mp.
27. transvascular aspiration catheter.mp. [mp=title, original title, abstract, name of substance word, subject heading word, unique identifier]
28. TVAC.mp. [mp=title, original title, abstract, name of substance word, subject heading word, unique identifier]
29. Pronto.mp. [mp=title, original title, abstract, name of substance word, subject heading word, unique identifier]
30. x-sizer.mp. [mp=title, original title, abstract, name of substance word, subject heading word, unique identifier]
31. angiojet.mp. [mp=title, original title, abstract, name of substance word, subject heading word, unique identifier]
32. filterwire.mp. [mp=title, original title, abstract, name of substance word, subject heading word, unique identifier]
33. spiderx.mp. [mp=title, original title, abstract, name of substance word, subject heading word, unique identifier]
34. spiderfx.mp. [mp=title, original title, abstract, name of substance word, subject heading word, unique identifier]
35. angioguard.mp. [mp=title, original title, abstract, name of substance word, subject heading word, unique identifier]
36. proxis.mp. [mp=title, original title, abstract, name of substance word, subject heading word, unique identifier]
37. interceptor plus.mp. [mp=title, original title, abstract, name of substance word, subject heading word, unique identifier]
38. rinspirator.mp. [mp=title, original title, abstract, name of substance word, subject heading word, unique identifier]
39. microvena trap.mp. [mp=title, original title, abstract, name of substance word, subject heading word, unique identifier]
40. percusurge.mp. [mp=title, original title, abstract, name of substance word, subject heading word, unique identifier]
41. triactiv.mp. [mp=title, original title, abstract, name of substance word, subject heading word, unique identifier]
42. cardioshield.mp. [mp=title, original title, abstract, name of substance word, subject heading word, unique identifier]
43. thrombobuster.mp. [mp=title, original title, abstract, name of substance word, subject heading word, unique identifier]
44. rio catheter.mp. [mp=title, original title, abstract, name of substance word, subject heading word, unique identifier]
45. fetch catheter.mp. [mp=title, original title, abstract, name of substance word, subject heading word, unique identifier]
46. quickcat.mp. [mp=title, original title, abstract, name of substance word, subject heading word, unique identifier]
47. rubicon catheter.mp. [mp=title, original title, abstract, name of substance word, subject heading word, unique identifier]
48. parodi anti-embolisation.mp. [mp=title, original title, abstract, name of substance word, subject heading word, unique identifier]
49. 18 or 19 or 20 or 21 or 22 or 23 or 24 or 25 or 26 or 27 or 28 or 29 or 30 or 31 or 32 or 33 or 34 or 35 or 36 or 37 or 38 or 39 or 40 or 41 or 42 or 43 or 44 or 45 or 46 or 47 or 48
50. 17 and 49
51. 50 not carotid.mp. [mp=title, original title, abstract, name of substance word, subject heading word, unique identifier]
52. limit 51 to humans
