# Supplementary material for: Systematic review: comparative effectiveness of adjunctive devices in patients with ST-segment elevation myocardial infarction undergoing percutaneous coronary intervention of native vessels
Source: BMC Cardiovasc Disord. 2011 Dec 20;11:74. doi: 10.1186/1471-2261-11-74 (PMC3313863; doi:10.1186/1471-2261-11-74)
Supplement: Additional file 2 — Table S1. Baseline study characteristics. This file contains an additional table with included trial baseline characteristics. [file 1471-2261-11-74-S2.DOC]

**Table S1**. Baseline study characteristics

| **Study, Year** | **Group** | **N** | **Mean Age (SD)** | **Male**  **(%)** | **TIMI-0/1 (%)** | **Mean Ischemic Time in Minutes (SD)^*^** | **Prior MI**  **(%)** | **Anterior  MI  (%)** | **Failed TL  (%)** | **IRA LAD (%)** | **Visible Lesion (%)** | **DM**  **(%)** | **HTN**  **(%)** | **HCL**  **(%)** | **Smoker**  **(%)** | **FHx**  **(%)** |
| --- | --- | --- | --- | --- | --- | --- | --- | --- | --- | --- | --- | --- | --- | --- | --- | --- |
| **Catheter aspiration devices** | |  |  |  |  |  |  |  |  |  |  |  |  |  |  |  |
| Dudek,  2010 | Diver CE  Control | 100  96 | 60.8 (10.2)  58.8 (10.3) | 80  81.7 | 96.9  97.9 | ---  --- | 2  0 | ---  --- | 0  0 | 39  39.6 | ---  --- | 13  9.6 | 58  54 | 43  48.5 | 63  63 | 33  26 |
| Liistro,  2009 | Export Thrombectomy Catheter  Control | 55  56 | 64 (11)  65 (11) | 78  77 | 69  76 | 189 (105)  209 (147) | 0  0 | ---  --- | 0  0 | 38  46 | ---  --- | 20  12 | 60  53 | 34  30 | 63    64 | 38    23 |
| Lipiecki,  2009 | Export Catheter  Control | 20  24 | 59 (13)  59 (13) | 60  75 | 100  95.83 | 426 (294)  444 (408) | 0  0 | ---  --- | 15  8 | 35  46 | ---  --- | 5  8 | 25  33 | 30  21 | 35  38 | ---  --- |
| Moura,  2009 | TAC  Control | 76  76 | ---  --- | ---  --- | ---  --- | ---  --- | ---  --- | ---  --- | ---  --- | ---  --- | ---  --- | ---  --- | ---  --- | ---  --- | ---  --- | ---  --- |
| Sardella,  2009 | Export Medtronic (EM)  Control | 88  87 | 66.7 (14.1)  64.6 (12.5) | 64.7  55.1 | 100  100 | 372 (54)  366 (108) | 0  0 | 43.18  42.53 | 0  0 | 43.2  43.7 | 100  100 | 23.8  18.4 | 67.0  49.4 | ---  --- | 48.8  26.4 | 29.5  36.8 |
| Chao,  2008 | Export Aspiration Catheter  Control | 37  37 | 60 (13)  62 (11) | 83.78  86.49 | ---  --- | 312 (183)  331 (175) | 11    3 | 60  65 | 0  0 | 56.76  59.46 | 81  73 | 32  22 | 57  57 | 60  57 | 41  46 | ---  --- |
| Chevalier,  2008 | Export Aspiration Catheter  Control | 120  129 | 59.2 (12.8)  61.2 (12.9) | 80.8  81.4 | 99.2  100 | 321.7 (413.5)  271.4 (197.6) | 10.83  10.85 | ---  --- | 0  0 | 47.5  51.9 | ---  --- | 16.7  13.2 | 41.4  44.2 | 36.7  41.9 | 42.5  35.7 | 32.5  25.6 |
| Ciszewski,  2008 | Rescue/Diver  Control | 65  70 | 64.3(12.4) ^†^ | 65.19 ^†^ | ---  --- | ---  --- | ---  --- | 32.28 ^†^ | 0  0 | ---  --- | ---  --- | ---  --- | ---  --- | ---  --- | ---  --- | ---  --- |
| Ikari,  2008 | TVAC  Control | 180  175 | 63.2 (10.6)  63.5 (9.9) | 80.6  77.7 | 74.6  75.3 | 270 (300)  312 (330) | ---  --- | ---  --- | 0  0 | 50.3  52.0 | 81.11  82.29 | 23.3  29.9 | 54.8  59.0 | 50.0  48.5 | 56.6  50.9 | 13.9  14.4 |
| Svilaas,  2008 | 6F Export Aspiration Catheter  Control | 535  536 | 63 (13)  63 (13) | 67.9  73.1 | 54.8  59.5 | 190 (110-270)^‡^  185 (107-263)^‡^ | 9.5  10.7 | ---  --- | 0  0 | 42.9  43.1 | 48.6^§^  44.0^§^ | 10.6  12.6 | 33.1  37.1 | 23.7  27.1 | 46.0  48.0 | 46.2  44.6 |
| DeLuca,  2006 | Diver CE  Control | 38  38 | 66.7 (14.1)  64.6 (12.5) | 71  55.3 | 100  100 | 432 (114)  456 (108) | 0  0 | ---  --- | 0  0 | 97.4  100 | 100  100 | 23.7  18.4 | 39.5  50 | ---  --- | 18.4  26.3 | 13.1  36.8 |
| Kaltoft,  2006 | Rescue Catheter  Control | 108  107 | 65 (11)  63 (13) | 76  80 | 68  69 | 242 (171-321)^‡^  208 (155-329)^‡^ | 13  10 | 46.30  42.99 | 0  0 | 46  43 | 69  79 | 8  6 | 31  21 | 9  9 | 55  64 | ---  --- |
| Lee,  2006 | Export Aspiration Catheter  Control | 67  66 | 60.8(1.05)^†^ | 69.9^†^ | ---  --- | ---  --- | ---  --- | ---  --- | 0  0 | 42.9^†^ | ---  --- | ---  --- | ---  --- | ---  --- | ---  --- | ---  --- |
| Silva-Orrego,  2006 | Pronto Extraction Catheter  Control | 74  74 | 57.3 (13)  58.9 (14) | 84  76 | 81  73 | 206 (115)  199 (124) | 0  0 | 42  51 | 0  0 | 43  51 | ---  --- | 21  15 | 37  46 | 34  25 | 54  60 | ---  --- |
| Burzotta,  2005 | Diver CE  Control | 50  49 | 61 (13)  60 (13) | 90.0  77.6 | 86  89.8 | 274 (137)  300 (202) | ---  --- | 40.0  51.0 | 32.0  24.5 | 40.0  51.0 | ---  --- | 22.0  18.4 | 62.0  57.1 | 54.0  34.7 | 62.0  53.1 | 30.0  22.4 |
| Noel,  2005 | Export  Control | 24  26 | 61.2(11.3)^†^ | ---  --- | 100||  100|| | 282 (186)^†^ | ---  --- | 44 ^†^ | ---  --- | ---  --- | ---  --- | ---  --- | ---  --- | ---  --- | ---  --- | ---  --- |
| Dudek,  2004 | Rescue System  Control | 40  32 | 56.7 (8.1)  59.1 (7.8) | 80  69 | 79  66 | 258 (198)  236 (162) | 15  25 | 40  56 | 0  0 | ---  --- | ---  --- | 10  19 | 75  81 | ---  --- | 45  31 | 40  50 |
| **Mechanical Thrombectomy Devices** | | |  |  |  |  |  |  |  |  |  |  |  |  |  |  |
| Migliorini  2010 | AngioJet Rheolytic Thrombectmy  Control | 256  245 | 63.0 (12.3)  64.3 (11.5) | 76  81 | 83.5  83.9 | 125 (85-221.5)^‡^  135 (86-227) ^‡^ | 3.9  4.9 | 39  37 | 0  0 | 42  37 | ---  --- | 14  15 | 47  47 | 30  35 | ---  --- | ---  --- |
| Ali,  2006 | AngioJet Catheter  Control | 240  240 | 60 (51.0-69.0)^‡^  59.9 (49.0  - 70.0)^‡^ | 75.8  74.2 | 68.4  63.2 | 144 (198)  150 (192) | ---  --- | ---  --- | 14.2  13.3 | 38.9  37.4 | 20.83  19.19 | 16.7  15.8 | 42.9  42.1 | 22.1  25.4 | 44.2  45.0 | ---  --- |
| Lefèvre  2005 | X-Sizer Catheter  Control | 100  101 | 61 (13)  62 (11) | 76  73 | 100  100 | 251 (151)  264 (194) | 10  6 | 54  50 | 0  0 | 55  48 | ---  --- | 25  18 | 54  50 | 58  61 | 52  51 | ---  --- |
| Antoniucci  2004 | AngioJet  Control | 50  50 | 63 (13)  66 (12) | 82  78 | 76  80 | 234 (120)  264 (168) | ---  --- | 34  46 | 0  0 | 34  46 | ---  --- | 18  16 | 36  38 | 46  48 | 38  28 | ---  --- |
| Napodano2003 | X-Sizer Catheter  Control | 46  46 | 61.3 (10.8)  63.6 (11.7) | 82.6  71.7 | 73.9  84.7 | 202.9 (204.9)  165.7 (134.7) | 17.4  6.5 | 39.1  43.5 | ---  --- | ---  --- | 100  100 | 13.0  13.0 | 60.9  65.2 | 50.0  52.1 | 45.6  34.8 | ---  --- |
| **Distal Filter Embolic Protection Devices** | | | |  |  |  |  |  |  |  |  |  |  |  |  |  |
| Ito,  2010 | Filtrap  Control | 19  17 | 62.7 (12)  63.7 (8.4) | 79  76 | 89  94 | 275 (223)  196 (223) | 0  0 | 100  100 | 0  0 | ---  --- | ---  --- | 47  29 | 58  53 | 74  59 | 68  71 | ---  --- |
| Kelbæk,  2008 | FilterWire-EZ or SpiderX protection device  Control | 312  314 | 62 (12.3)  63 (12.1) | 74.4  72.0 | 67  68 | 200 (26-1350) ^‡^  199 (40-996) ^‡^ | 6.4  6.4 | ---  --- | 0  0 | 44  38 | 68  75 | 9.0  11.8 | 32.1  34.1 | 18.6  20.4 | 56.7  50.3 | 36.5  37.6 |
| Cura,  2007 | SpideRX  Control | 70  70 | 60.2 (9.9)  60.4 (10.4) | 86  77 | 85  83 | 150 (80-270) ^‡^  146 (75-236) ^‡^ | 21  13 | ---  --- | 3  4 | 53  56 | 90  97 | 19  20 | 56  49 | ---  --- | 33  47 | ---  --- |
| Guetta, 2007 | FilterWire EZ  Control | 51  49 | 60 (12)  57 (10) | 82  82 | 78  92 | 180 (90-420) ^‡^  120 (66-180) ^‡^ | 12  11 | ---  --- | ---  --- | 51  53 | ---  --- | 22  23 | 44  51 | 48  50 | 43  44 | ---  --- |
| Lefèvre,  2004 | AngioGuardXP  Control | 32  28 | 61 (15)  62 (12) | 81  83 | 71.88  67.86 | ---  --- | ---  --- | ---  --- | ---  --- | 41  53 | 100  100 | 19  14 | 50  43 | 62  61 | ---  --- | ---  --- |
| **Distal Balloon Embolic Protection Devices** | | | |  |  |  |  |  |  |  |  |  |  |  |  |  |
| Duan,  2010 | PercuSurge Guardwire Plus  Control | 46  50 | 55 (7)  56 (8) | 86.96  82 | 80.4  80.0 | 289 (58)  282 (60) | ---  --- | 100  100 | 0  0 | 100  100 | ---  --- | 6.5  8.0 | 17.4  20.0 | 15.2  20.0 | 63.0  58.0 | ---  --- |
| Pan,  2010 | PercuSurge Guardwire  Control | 52  52 | 67 (6.1)† | 61.54† | 73.1  71.1 | 157 (47)  161 (43) | 0  0 | 40.4† | ---  --- | 55.8  51.9 | ---  --- | 32.7  30.7 | 55.8  51.9 | ---  --- | 42.3  46.2 | ---  --- |
| Tahk,  2008 | PercuSurge GuardWire  Control | 60  56 | 55.9 (13.9)  58.8 (14.5) | 85  71 | 67  76 | 339.3 (189.2)  327.8 (209.5) | 0  0 | 53  56 | 0  0 | 53  56 | ---  --- | 20  21 | 36  54 | ---  --- | 68  57 | ---  --- |
| Hahn,  2007 | GuardWire  Control | 19  20 | 55 (45-62)^‡^  56 (45-65)^‡^ | 79  95 | 95  75 | 212 (160-325)‡  248 (185-480)^‡^ | ---  --- | 58  55 | 0  0 | 58  55 | ---  --- | 32  15 | 47  25 | 26  20 | 63  65 | ---  --- |
| Matsuo,  2007 | GuardWire Distal Protection System  Control | 80  74 | 65 (12)  65 (13) | 86  76 | 78  71 | 312 (252)  264 (174) | 5  8 | ---  --- | ---  --- | 57  40 | ---  --- | 20  25 | 51  49 | 35  39 | 47  50 | 12    7 |
| Muramatsu, 2007 | GuardWire Plus System  Control | 173  168 | 63.5 (12.3)  64.7 (11.1) | 78.6  72.9 | 69  68 | 252 (168)  264 (204) | 1.7  2.9 | ---  --- | 0  0 | 50  48 | ---  --- | 31.8  32.3 | 42.2  44.1 | 32.9  32.9 | 51.4  49.4 | 4.6  4.1 |
| Zhou,  2007 | PercuSurge GuardWire  Control | 52  60 | 55 (14)  57 (15) | 62  67 | 100  100 | 310 (145)  315 (176) | ---  --- | ---  --- | 0  0 | 54  48 | ---  --- | 23  22 | 37  35 | ---  --- | 67  60 | ---  --- |
| Okamura,  2005 | PercuSurge GuardWire  Control | 8  8 | 59 (13)  59 (8) | 75  88 | 75  38 | 450 (348)  510 (492 | ---  --- | ---  --- | ---  --- | 25  75 | ---  --- | 25  25 | 38  63 | 38  38 | 75  63 | ---  --- |
| Stone,  2005 | GuardWire Plus  Control | 252  249 | 58.5(51.1-69.3)‡  59.8 (52.1-69.3)^‡^ | 76.2  80.7 | 64.0  67.8 | 233 (178-296)^‡^  211 (158-273)^‡^ | 9.5  12.4 | ---  --- | 18.3  18.9 | 40.2  38.5 | 72.1  72.1 | 7.5  17.3 | 35.9  38.2 | 20.2  28.5 | 40.5  44.6 | ---  --- |
| **Proximal Balloon Embolic Protection Devices** | | | |  |  |  |  |  |  |  |  |  |  |  |  |  |
| Haeck,  2009 | Proxis  Control | 141  143 | 62 (11)  59 (11) | 80  80 | 98.58  96.50 | 170 (132-234)^‡^  153 (126-212)^‡^ | 6  9 | ---  --- | 0  0 | 29  29 | 76  66 | 12  6 | 31  23 | 21  13 | 50  65 | 35  38 |

*Symptom onset to balloon, ischemic time, symptom to randomization,symptom onset to hospital, symptom onset to laboratory, symptom onset to angiography, symptom onset to admission, symptom onset to procedure, symptom onset to emergency room, time to treatment, symptom onset to stenting, symptom onset to hospital arrival, symptom onset to reperfusion, elapsed time before reperfusion

^†^Mean for the total study population

^‡^Median (interquartile range)

^§^% of visible thrombi out of all thrombi

^||^TIMI<3

Abbreviations: DM=diabetes mellitus; FHx=family history; HCL=hypercholesterolemia; HTN=hypertension; IRA=infarct-related artery; LAD=left anterior descending artery; MI=myocardial infarction; N=number of participants in the group; SD=standard deviation; TAC=thrombectomy aspiration catheter; TIMI=thrombolysis in myocardial infarction; TL=thrombolysis; TVAC = transvascular aspiration catheter
