# Supplementary material for: Systematic review: comparative effectiveness of adjunctive devices in patients with ST-segment elevation myocardial infarction undergoing percutaneous coronary intervention of native vessels
Source: BMC Cardiovasc Disord. 2011 Dec 20;11:74. doi: 10.1186/1471-2261-11-74 (PMC3313863; doi:10.1186/1471-2261-11-74)
Supplement: Additional file 3 — Impact of catheter aspiration devices versus control on mortality using the maximal duration of followup in patients with ST-segment elevation myocardial infarction. Figure of the Impact of catheter aspiration devices versus control on mortality using the maximal duration of followup in patients with ST-segment elevation myocardial infarction. The squares represent individual point estimates. The size of the square represents the weight given to each study in the meta-analysis. Horizontal lines through each square represent 95 percent confidence intervals. The diamond represents the combined results. The solid vertical line extending from 1 is the null value. [file 1471-2261-11-74-S3.DOC]

*0.1*

*0.2*

*0.5*

*1*

*2*

*5*

*10*

*100*

*Noel, 2005*

*0.36 (0.00, 4.03)*

*Burzotta, 2005*

*1.00 (0.24, 4.16)*

*Silva-Orrego, 2006*

** (excluded)*

*Kaltoft, 2006*

*0.33 (0.00, 3.78)*

*De Luca, 2006*

*0.70 (0.16, 2.95)*

*Svilaas, 2008*

*0.61 (0.38, 0.98)*

*Ikari, 2008*

*1.86 (0.25, 14.12)*

*Chevalier, 2008*

*0.86 (0.25, 2.89)*

*Chao, 2008*

*2.76 (0.24, infinity)*

*Sardella, 2009*

*0.11 (0.00, 0.93)*

*Dudek, 2010*

*1.28 (0.33, 5.01)*

*combined [random]*

*0.69 (0.47, 1.02)*

*relative risk (95% confidence interval)*

Cochran Q: P=0.870

I²: 0 percent

Egger: P=0.638
