# Supplementary material for: Systematic review: comparative effectiveness of adjunctive devices in patients with ST-segment elevation myocardial infarction undergoing percutaneous coronary intervention of native vessels
Source: BMC Cardiovasc Disord. 2011 Dec 20;11:74. doi: 10.1186/1471-2261-11-74 (PMC3313863; doi:10.1186/1471-2261-11-74)
Supplement: Additional file 4 — Impact of mechanical thrombectomy devices versus control on mortality using the maximal duration of followup in patients with ST-segment elevation myocardial infarction. Figure of the Impact of mechanical thrombectomy devices versus control on mortality using the maximal duration of followup in patients with ST-segment elevation myocardial infarction. The squares represent individual point estimates. The size of the square represents the weight given to each study in the meta-analysis. Horizontal lines through each square represent 95 percent confidence intervals. The diamond represents the combined results. The solid vertical line extending from 1 is the null value. [file 1471-2261-11-74-S4.DOC]

*0.2*

*0.5*

*1*

*2*

*5*

*10*

*Napodano, 2003*

*1.00 (0.24, 4.16)*

*Antoniucci, 2004*

** (excluded)*

*Lefèvre, 2005*

*1.52 (0.47, 4.88)*

*Ali, 2006*

*2.80 (1.07, 7.38)*

*Migliorini, 2010*

*0.50 (0.21, 1.17)*

*combined [random]*

*1.19 (0.51, 2.76)*

*relative risk (95% confidence interval)*

I²: 54.9 percent

Egger: P=0.736
