# Supplementary material for: Systematic review: comparative effectiveness of adjunctive devices in patients with ST-segment elevation myocardial infarction undergoing percutaneous coronary intervention of native vessels
Source: BMC Cardiovasc Disord. 2011 Dec 20;11:74. doi: 10.1186/1471-2261-11-74 (PMC3313863; doi:10.1186/1471-2261-11-74)
Supplement: Additional file 6 — Impact of distal balloon embolic protection devices versus control on mortality using the maximal duration of followup versus control in patients with ST-segment elevation myocardial infarction. Figure of the Impact of distal balloon embolic protection devices versus control on mortality using the maximal duration of followup versus control in patients with ST-segment elevation myocardial infarction. The squares represent individual point estimates. The size of the square represents the weight given to each study in the meta-analysis. Horizontal lines through each square represent 95 percent confidence intervals. The diamond represents the combined results. The solid vertical line extending from 1 is the null value. [file 1471-2261-11-74-S6.DOC]

*0.2*

*0.5*

*1*

*2*

*5*

*Stone, 2005*

*0.96 (0.38, 2.43)*

*Muramatsu, 2007*

*0.97 (0.44, 2.14)*

*Hahn, 2007*

*0.12 (0.00, 0.91)*

*Tahk, 2008*

*0.19 (0.00, 1.81)*

*combined [random]*

*0.82 (0.45, 1.51)*

*relative risk (95% confidence interval)*

Cochran Q: P=0.380

I²: 2.5 percent

Egger: P=0.023
