# Supplementary material for: Systematic review: comparative effectiveness of adjunctive devices in patients with ST-segment elevation myocardial infarction undergoing percutaneous coronary intervention of native vessels
Source: BMC Cardiovasc Disord. 2011 Dec 20;11:74. doi: 10.1186/1471-2261-11-74 (PMC3313863; doi:10.1186/1471-2261-11-74)
Supplement: Additional file 7 — Impact of embolic protection devices combined versus control on mortality using the maximal duration of followup in patients with ST- segment elevation myocardial infarction. Figure of the Impact of embolic protection devices combined versus control on mortality using the maximal duration of followup in patients with ST-segment elevation myocardial infarction. The squares represent individual point estimates. The size of the square represents the weight given to each study in the meta-analysis. Horizontal lines through each square represent 95 percent confidence intervals. The diamond represents the combined results. The solid vertical line extending from 1 is the null value. [file 1471-2261-11-74-S7.DOC]

*0.01*

*0.1*

*0.2*

*0.5*

*1*

*2*

*5*

*10*

*Lefevre, 2004*

*0.88 (0.09, 8.17)*

*Stone, 2005*

*0.96 (0.38, 2.43)*

*Muramatsu, 2007*

*0.97 (0.44, 2.14)*

*Hahn, 2007*

*0.12 (0.00, 0.91)*

*Guetta, 2007*

*4.81 (0.51, infinity)*

*Cura, 2007*

*1.25 (0.38, 4.16)*

*Tahk, 2008*

*0.19 (0.00, 1.81)*

*Kelbaek, 2008*

*0.87 (0.43, 1.78)*

*Haeck, 2009*

*0.51 (0.11, 2.33)*

*Ito, 2010*

*0.30 (0.00, 3.30)*

*combined [random]*

*0.87 (0.58, 1.30)*

*relative risk (95% confidence interval)*

Cochran Q: P=0.798

I²: 0 percent

Egger: P=0.254
