# Supplementary material for: Systematic review: comparative effectiveness of adjunctive devices in patients with ST-segment elevation myocardial infarction undergoing percutaneous coronary intervention of native vessels
Source: BMC Cardiovasc Disord. 2011 Dec 20;11:74. doi: 10.1186/1471-2261-11-74 (PMC3313863; doi:10.1186/1471-2261-11-74)
Supplement: Additional file 8 — Impact of catheter aspiration devices versus control on myocardial infarction using the maximal duration of followup in patients with ST-segment elevation myocardial infarction. Figure of the Impact of catheter aspiration devices versus control on myocardial infarction using the maximal duration of followup in patients with ST-segment elevation myocardial infarction. The squares represent individual point estimates. The size of the square represents the weight given to each study in the meta-analysis. Horizontal lines through each square represent 95 percent confidence intervals. The diamond represents the combined results. The solid vertical line extending from 1 is the null value. [file 1471-2261-11-74-S8.DOC]

*0.01*

*0.1*

*0.2*

*0.5*

*1*

*2*

*5*

*10*

*100*

*Burzotta, 2005*

*1.00 (0.18, 5.50)*

*Silva-Orrego, 2006*

*0.32 (0.00, 3.60)*

*Kaltoft, 2006*

*0.33 (0.00, 3.78)*

*De Luca, 2006*

*3.25 (0.29, infinity)*

*Svilaas, 2008*

*0.52 (0.27, 1.03)*

*Ikari, 2008*

*0.31 (0.00, 3.55)*

*Chevalier, 2008*

*2.15 (0.28, 16.30)*

*Sardella, 2009*

*0.33 (0.00, 3.76)*

*Liistro, 2009*

*1.02 (0.24, 4.26)*

*Dudek, 2010*

*0.32 (0.05, 2.19)*

*combined [random]*

*0.61 (0.36, 1.04)*

*relative risk (95% confidence interval)*

Cochran Q: P=0.915

I²: 0 percent

Egger: P=0.651
