# Supplementary material for: Systematic review: comparative effectiveness of adjunctive devices in patients with ST-segment elevation myocardial infarction undergoing percutaneous coronary intervention of native vessels
Source: BMC Cardiovasc Disord. 2011 Dec 20;11:74. doi: 10.1186/1471-2261-11-74 (PMC3313863; doi:10.1186/1471-2261-11-74)
Supplement: Additional file 9 — Impact of mechanical thrombectomy devices versus control on myocardial infarction using the maximal duration of followup in patients with ST-segment elevation myocardial infarction. Figure of the Impact of mechanical thrombectomy devices versus control on myocardial infarction using the maximal duration of followup in patients with ST-segment elevation myocardial infarction. The squares represent individual point estimates. The size of the square represents the weight given to each study in the meta-analysis. Horizontal lines through each square represent 95 percent confidence intervals. The diamond represents the combined results. The solid vertical line extending from 1 is the null value. [file 1471-2261-11-74-S9.DOC]

*0.1*

*0.2*

*0.5*

*1*

*2*

*5*

*Napodano, 2003*

*1.00 (0.24, 4.16)*

*Antoniucci, 2004*

** (excluded)*

*Lefèvre, 2005*

*0.51 (0.11, 2.31)*

*Ali, 2006*

** (excluded)*

*Migliorini, 2010*

*0.66 (0.13, 3.29)*

*combined [random]*

*0.71 (0.27, 1.85)*

*relative risk (95% confidence interval)*

Cochran Q: P=0.838

I²: 0 percent

Egger: Too few strata
