# Supplementary material for: Systematic review: comparative effectiveness of adjunctive devices in patients with ST-segment elevation myocardial infarction undergoing percutaneous coronary intervention of native vessels
Source: BMC Cardiovasc Disord. 2011 Dec 20;11:74. doi: 10.1186/1471-2261-11-74 (PMC3313863; doi:10.1186/1471-2261-11-74)
Supplement: Additional file 10 — Impact of distal filter embolic protection devices versus control on myocardial infarction using the maximal duration of followup in patients with ST-segment elevation myocardial infarction. Figure of the Impact of distal filter embolic protection devices versus control on myocardial infarction using the maximal duration of followup in patients with ST-segment elevation myocardial infarction. The squares represent individual point estimates. The size of the square represents the weight given to each study in the meta-analysis. Horizontal lines through each square represent 95 percent confidence intervals. The diamond represents the combined results. The solid vertical line extending from 1 is the null value. [file 1471-2261-11-74-S10.DOC]

*0.01*

*0.1*

*0.2*

*0.5*

*1*

*2*

*5*

*10*

*Lefevre, 2004*

*0.88 (0.09, 8.17)*

*Guetta, 2007*

*0.32 (0.00, 3.63)*

*Cura, 2007*

*0.09 (0.00, 0.74)*

*Kelbaek, 2008*

*2.35 (0.67, 8.28)*

*Ito, 2010*

** (excluded)*

*combined [random]*

*0.72 (0.15, 3.34)*

*relative risk (95% confidence interval)*

Cochran Q: P=0.173

I²: 39.8 percent

Egger: P=0.128
