# Supplementary material for: Systematic review: comparative effectiveness of adjunctive devices in patients with ST-segment elevation myocardial infarction undergoing percutaneous coronary intervention of native vessels
Source: BMC Cardiovasc Disord. 2011 Dec 20;11:74. doi: 10.1186/1471-2261-11-74 (PMC3313863; doi:10.1186/1471-2261-11-74)
Supplement: Additional file 11 — Impact of distal balloon embolic protection devices versus control on myocardial infarction using the maximal duration of followup in patients with ST-segment elevation myocardial infarction. Figure of the Impact of distal balloon embolic protection devices versus control on myocardial infarction using the maximal duration of followup in patients with ST-segment elevation myocardial infarction. The squares represent individual point estimates. The size of the square represents the weight given to each study in the meta-analysis. Horizontal lines through each square represent 95 percent confidence intervals. The diamond represents the combined results. The solid vertical line extending from 1 is the null value. [file 1471-2261-11-74-S11.DOC]

*0.1*

*0.2*

*0.5*

*1*

*2*

*5*

*10*

*Stone, 2005*

*0.64 (0.24, 1.70)*

*Muramatsu, 2007*

*0.32 (0.00, 3.71)*

*Matsuo, 2007*

*2.78 (0.24, infinity)*

*Hahn, 2007*

*0.35 (0.00, 3.88)*

*Tahk, 2008*

*0.96 (0.10, 9.09)*

*combined [random]*

*0.67 (0.29, 1.57)*

*relative risk (95% confidence interval)*

Cochran Q: P=0.877

I²: 0 percent

Egger: P=0.820
