# Supplementary material for: Systematic review: comparative effectiveness of adjunctive devices in patients with ST-segment elevation myocardial infarction undergoing percutaneous coronary intervention of native vessels
Source: BMC Cardiovasc Disord. 2011 Dec 20;11:74. doi: 10.1186/1471-2261-11-74 (PMC3313863; doi:10.1186/1471-2261-11-74)
Supplement: Additional file 13 — Impact of catheter aspiration devices versus control on stroke using the maximal duration of followup in patients with ST-segment elevation myocardial infarction. Figure of the Impact of catheter aspiration devices versus control on stroke using the maximal duration of followup in patients with ST-segment elevation myocardial infarction. The squares represent individual point estimates. The size of the square represents the weight given to each study in the meta-analysis. Horizontal lines through each square represent 95 percent confidence intervals. The diamond represents the combined results. The solid vertical line extending from 1 is the null value. [file 1471-2261-11-74-S13.DOC]

*0.1*

*0.2*

*0.5*

*1*

*2*

*5*

*10*

*100*

*Burzotta, 2005*

*1.00 (0.11, 9.42)*

*Silva-Orrego, 2006*

** (excluded)*

*Kaltoft, 2006*

*4.95 (0.52, infinity)*

*Chevalier, 2008*

*5.37 (0.57, infinity)*

*Sardella, 2009*

*4.94 (0.52, infinity)*

*combined [random]*

*3.18 (0.73, 13.88)*

*relative risk (95% confidence interval)*

Cochran Q: P=0.807

I²: 0 percent

Egger: P=0.001
