# Supplementary material for: Systematic review: comparative effectiveness of adjunctive devices in patients with ST-segment elevation myocardial infarction undergoing percutaneous coronary intervention of native vessels
Source: BMC Cardiovasc Disord. 2011 Dec 20;11:74. doi: 10.1186/1471-2261-11-74 (PMC3313863; doi:10.1186/1471-2261-11-74)
Supplement: Additional file 15 — Impact of embolic protection devices combined versus control on stroke using the maximal duration of followup in patients with ST-segment elevation myocardial infarction. Figure of the Impact of embolic protection devices combined versus control on stroke using the maximal duration of followup in patients with ST-segment elevation myocardial infarction. The squares represent individual point estimates. The size of the square represents the weight given to each study in the meta-analysis. Horizontal lines through each square represent 95 percent confidence intervals. The diamond represents the combined results. The solid vertical line extending from 1 is the null value. [file 1471-2261-11-74-S15.DOC]

*0.1*

*0.2*

*0.5*

*1*

*2*

*5*

*10*

*Stone, 2005*

*0.48 (0.10, 2.22)*

*Kelbaek, 2008*

*1.51 (0.30, 7.52)*

*Haeck, 2009*

*0.20 (0.00, 1.93)*

*combined [random]*

*0.68 (0.22, 2.11)*

*relative risk (95% confidence interval)*

Cochran Q: P=0.459

I²: 0 percent

Egger: Too few strata
