# Supplementary material for: Systematic review: comparative effectiveness of adjunctive devices in patients with ST-segment elevation myocardial infarction undergoing percutaneous coronary intervention of native vessels
Source: BMC Cardiovasc Disord. 2011 Dec 20;11:74. doi: 10.1186/1471-2261-11-74 (PMC3313863; doi:10.1186/1471-2261-11-74)
Supplement: Additional file 16 — Impact of catheter aspiration devices versus control on target revascularization using the maximal duration of followup in patients with ST-segment elevation myocardial infarction. Figure of the Impact of catheter aspiration devices versus control on target revascularization using the maximal duration of followup in patients with ST-segment elevation myocardial infarction. The squares represent individual point estimates. The size of the square represents the weight given to each study in the meta-analysis. Horizontal lines through each square represent 95 percent confidence intervals. The diamond represents the combined results. The solid vertical line extending from 1 is the null value. [file 1471-2261-11-74-S16.DOC]

*0.01*

*0.1*

*0.2*

*0.5*

*1*

*2*

*5*

*10*

*100*

*Burzotta, 2005*

*1.00 (0.11, 9.42)*

*Silva-Orrego, 2006*

*0.47 (0.06, 3.54)*

*Svilaas, 2008*

*0.87 (0.63, 1.20)*

*Ikari, 2008*

*0.60 (0.36, 1.00)*

*Chevalier, 2008*

*2.15 (0.28, 16.30)*

*Chao, 2008*

*0.75 (0.20, 2.82)*

*Sardella, 2009*

*0.79 (0.24, 2.64)*

*Liistro, 2009*

*1.02 (0.29, 3.56)*

*Dudek, 2010*

*0.32 (0.00, 3.66)*

*combined [random]*

*0.79 (0.61, 1.02)*

*relative risk (95% confidence interval)*

Cochran Q: P=0.948

I²: 0 percent

Egger: P=0.885
