# Supplementary material for: Systematic review: comparative effectiveness of adjunctive devices in patients with ST-segment elevation myocardial infarction undergoing percutaneous coronary intervention of native vessels
Source: BMC Cardiovasc Disord. 2011 Dec 20;11:74. doi: 10.1186/1471-2261-11-74 (PMC3313863; doi:10.1186/1471-2261-11-74)
Supplement: Additional file 20 — Impact of embolic protection devices combined versus control on target revascularization using the maximal duration of followup in patients with ST-segment elevation myocardial infarction. Figure of the Impact of embolic protection devices combined versus control on target revascularization using the maximal duration of followup in patients with ST-segment elevation myocardial infarction. The squares represent individual point estimates. The size of the square represents the weight given to each study in the meta-analysis. Horizontal lines through each square represent 95 percent confidence intervals. The diamond represents the combined results. The solid vertical line extending from 1 is the null value. [file 1471-2261-11-74-S20.DOC]

*0.1*

*0.2*

*0.5*

*1*

*2*

*5*

*10*

*Stone, 2005*

*1.11 (0.55, 2.24)*

*Muramatsu, 2007*

*1.03 (0.55, 1.95)*

*Matsuo, 2007*

*0.51 (0.19, 1.39)*

*Hahn, 2007*

*0.21 (0.00, 1.89)*

*Cura, 2007*

*1.00 (0.35, 2.82)*

*Tahk, 2008*

*1.44 (0.30, 7.04)*

*Kelbaek, 2008*

*1.78 (1.09, 2.93)*

*Haeck, 2009*

*0.71 (0.29, 1.75)*

*Ito, 2010*

** (excluded)*

*combined [random]*

*1.11 (0.80, 1.52)*

*relative risk (95% confidence interval)*

Cochran Q: P=0.353

I²: 10 percent

Egger: P=0.066
