# Supplementary material for: Systematic review: comparative effectiveness of adjunctive devices in patients with ST-segment elevation myocardial infarction undergoing percutaneous coronary intervention of native vessels
Source: BMC Cardiovasc Disord. 2011 Dec 20;11:74. doi: 10.1186/1471-2261-11-74 (PMC3313863; doi:10.1186/1471-2261-11-74)
Supplement: Additional file 21 — Impact of catheter aspiration devices versus control on MACE of maximal duration of followup in patients with ST-segment elevation myocardial infarction. Figure of the Impact of catheter aspiration devices versus control on MACE of maximal duration of followup in patients with ST-segment elevation myocardial infarction. The squares represent individual point estimates. The size of the square represents the weight given to each study in the meta-analysis. Horizontal lines through each square represent 95 percent confidence intervals. The diamond represents the combined results. The solid vertical line extending from 1 is the null value. [file 1471-2261-11-74-S21.DOC]

*0.01*

*0.1*

*0.2*

*0.5*

*1*

*2*

*5*

*10*

*Noel, 2005*

*0.54 (0.07, 3.91)*

*Burzotta, 2005*

*1.00 (0.33, 3.05)*

*Kaltoft, 2006*

*0.99 (0.18, 5.54)*

*De Luca, 2006*

*0.81 (0.21, 3.05)*

*Svilaas, 2008*

*0.82 (0.64, 1.05)*

*Ikari, 2008*

*0.56 (0.40, 0.80)*

*Chevalier, 2008*

*1.25 (0.45, 3.47)*

*Chao, 2007*

*0.50 (0.19, 1.26)*

*Sardella, 2009*

*0.33 (0.12, 0.93)*

*Liistro, 2009*

*1.16 (0.47, 2.91)*

*Dudek, 2010*

*0.80 (0.27, 2.40)*

*combined [random]*

*0.73 (0.61, 0.88)*

*relative risk (95% confidence interval)*

Cochran Q: P=0.645

I²: 0 percent

Egger: P=0.965
