# Supplementary material for: Systematic review: comparative effectiveness of adjunctive devices in patients with ST-segment elevation myocardial infarction undergoing percutaneous coronary intervention of native vessels
Source: BMC Cardiovasc Disord. 2011 Dec 20;11:74. doi: 10.1186/1471-2261-11-74 (PMC3313863; doi:10.1186/1471-2261-11-74)
Supplement: Additional file 23 — Impact of distal filter embolic protection devices versus control on MACE using the maximal duration of followup in patients with ST-segment elevation myocardial infarction. Figure of the Impact of distal filter embolic protection devices versus control on MACE using the maximal duration of followup in patients with ST-segment elevation myocardial infarction. The squares represent individual point estimates. The size of the square represents the weight given to each study in the meta-analysis. Horizontal lines through each square represent 95 percent confidence intervals. The diamond represents the combined results. The solid vertical line extending from 1 is the null value. [file 1471-2261-11-74-S23.DOC]

*0.1*

*0.2*

*0.5*

*1*

*2*

*5*

*10*

*100*

*Lefevre, 2004*

*0.88 (0.16, 4.74)*

*Guetta, 2007*

*2.88 (0.43, 19.79)*

*Cura, 2007*

*0.91 (0.42, 1.96)*

*Kelbaek, 2008*

*1.48 (1.03, 2.15)*

*Ito, 2010*

*0.30 (0.00, 3.30)*

*combined [random]*

*1.34 (0.97, 1.86)*

*relative risk (95% confidence interval)*

Cochran Q: P=0.601

I²: 0 percent

Egger: P=0.419
