# Supplementary material for: Systematic review: comparative effectiveness of adjunctive devices in patients with ST-segment elevation myocardial infarction undergoing percutaneous coronary intervention of native vessels
Source: BMC Cardiovasc Disord. 2011 Dec 20;11:74. doi: 10.1186/1471-2261-11-74 (PMC3313863; doi:10.1186/1471-2261-11-74)
Supplement: Additional file 27 — Impact of mechanical thrombectomy devices versus control on ST-segment resolution in patients with ST-segment elevation myocardial infarction Figure of the Impact of mechanical thrombectomy devices versus control on ST-segment resolution in patients with ST-segment elevation myocardial infarction. The squares represent individual point estimates. The size of the square represents the weight given to each study in the meta-analysis. Horizontal lines through each square represent 95 percent confidence intervals. The diamond represents the combined results. The solid vertical line extending from 1 is the null value. [file 1471-2261-11-74-S27.DOC]

*0.5*

*1*

*2*

*5*

*10*

*100*

*Dudek, 2004*

*2.70 (1.51, 5.24)*

*Noel, 2005*

*4.33 (1.55, 13.21)*

*Burzotta, 2005*

*1.72 (1.13, 2.68)*

*Silva-Orrego, 2006*

*1.35 (1.03, 1.80)*

*Lee, 2006*

*1.64 (1.14, 2.42)*

*Kaltoft, 2006*

*1.04 (0.72, 1.50)*

*De Luca, 2006*

*1.48 (1.08, 2.10)*

*Svilaas, 2008*

*1.28 (1.13, 1.45)*

*Ikari, 2008*

*1.21 (0.80, 1.83)*

*Chevalier, 2008*

*1.13 (0.95, 1.34)*

*Sardella, 2009*

*2.10 (1.60, 2.84)*

*Moura, 2009*

*2.03 (1.58, 2.71)*

*Lipiecki, 2009*

*1.11 (0.62, 1.97)*

*Liistro, 2009*

*1.80 (1.27, 2.65)*

*Dudek, 2010*

*1.53 (1.11, 2.14)*

*combined [random]*

*1.51 (1.32, 1.73)*

*relative risk (95% confidence interval)*

Cochran Q: P=0.003

I²: 75.1 percent

Egger: P=0.402
