# Supplementary material for: Systematic review: comparative effectiveness of adjunctive devices in patients with ST-segment elevation myocardial infarction undergoing percutaneous coronary intervention of native vessels
Source: BMC Cardiovasc Disord. 2011 Dec 20;11:74. doi: 10.1186/1471-2261-11-74 (PMC3313863; doi:10.1186/1471-2261-11-74)
Supplement: Additional file 31 — Impact of catheter aspiration devices versus control on myocardial blush grade of 3 in patients with ST-segment elevation myocardial infarction. Figure of the Impact of catheter aspiration devices versus control on myocardial blush grade of 3 in patients with ST-segment elevation myocardial infarction. The squares represent individual point estimates. The size of the square represents the weight given to each study in the meta-analysis. Horizontal lines through each square represent 95 percent confidence intervals. The diamond represents the combined results. The solid vertical line extending from 1 is the null value. [file 1471-2261-11-74-S31.DOC]

*0.2*

*0.5*

*1*

*2*

*5*

*10*

*Dudek, 2004*

*1.47 (0.89, 2.55)*

*Burzotta, 2005*

*1.87 (1.04, 3.48)*

*Silva-Orrego, 2006*

*2.03 (1.58, 2.72)*

*Lee, 2006*

*1.97 (0.93, 4.25)*

*De Luca, 2006*

*2.80 (1.18, 6.95)*

*Svilaas, 2008*

*1.42 (1.21, 1.67)*

*Ikari, 2008*

*2.25 (1.62, 3.16)*

*Chevalier, 2008*

*1.40 (0.96, 2.05)*

*Sardella, 2009*

*2.45 (1.74, 3.55)*

*Moura, 2009*

*1.45 (1.21, 1.79)*

*Lipiecki, 2009*

*1.03 (0.42, 2.49)*

*Liistro, 2009*

*1.30 (1.10, 1.60)*

*Dudek, 2010*

*1.32 (1.07, 1.66)*

*combined [random]*

*1.61 (1.41, 1.84)*

*relative risk (95% confidence interval)*

Cochran Q: P=0.008

I²: 55.4 percent

Egger: P=0.117
