# Supplementary material for: Systematic review: comparative effectiveness of adjunctive devices in patients with ST-segment elevation myocardial infarction undergoing percutaneous coronary intervention of native vessels
Source: BMC Cardiovasc Disord. 2011 Dec 20;11:74. doi: 10.1186/1471-2261-11-74 (PMC3313863; doi:10.1186/1471-2261-11-74)
Supplement: Additional file 32 — Impact of mechanical thrombectomy devices versus control on myocardial blush grade of 3 in patients with ST-segment elevation myocardial infarction. Figure of the Impact of mechanical thrombectomy devices versus control on myocardial blush grade of 3 in patients with ST-segment elevation myocardial infarction. The squares represent individual point estimates. The size of the square represents the weight given to each study in the meta-analysis. Horizontal lines through each square represent 95 percent confidence intervals. The diamond represents the combined results. The solid vertical line extending from 1 is the null value. [file 1471-2261-11-74-S32.DOC]

*0.5*

*1*

*2*

*5*

*Napodano, 2003*

*1.94 (1.31, 3.02)*

*Lefèvre, 2005*

*1.02 (0.67, 1.57)*

*Ali, 2006*

*0.84 (0.64, 1.12)*

*Migliorini, 2010*

*0.91 (0.82, 1.01)*

*combined [random]*

*1.07 (0.80, 1.43)*

*relative risk (95% confidence interval)*

Cochran Q: P=0.005

I²: 76.5 percent

Egger: P=0.408
