# Supplementary material for: Systematic review: comparative effectiveness of adjunctive devices in patients with ST-segment elevation myocardial infarction undergoing percutaneous coronary intervention of native vessels
Source: BMC Cardiovasc Disord. 2011 Dec 20;11:74. doi: 10.1186/1471-2261-11-74 (PMC3313863; doi:10.1186/1471-2261-11-74)
Supplement: Additional file 35 — Impact of embolic protection devices combined versus control on myocardial blush grade of 3 in patients with ST-segment elevation myocardial infarction. Figure of the Impact of embolic protection devices combined versus control on myocardial blush grade of 3 in patients with ST-segment elevation myocardial infarction. The squares represent individual point estimates. The size of the square represents the weight given to each study in the meta-analysis. Horizontal lines through each square represent 95 percent confidence intervals. The diamond represents the combined results. The solid vertical line extending from 1 is the null value. [file 1471-2261-11-74-S35.DOC]

*0.2*

*0.5*

*1*

*2*

*5*

*Stone, 2005*

*1.16 (0.98, 1.36)*

*Zhou, 2007*

*1.96 (1.32, 2.99)*

*Muramatsu, 2007*

*1.24 (0.83, 1.86)*

*Matsuo, 2007*

*1.33 (0.97, 1.85)*

*Hahn, 2007*

*1.26 (0.48, 3.39)*

*Guetta, 2007*

*1.01 (0.76, 1.35)*

*Cura, 2007*

*0.94 (0.75, 1.18)*

*Tahk, 2008*

*1.82 (1.25, 2.75)*

*Haeck, 2009*

*0.98 (0.87, 1.10)*

*combined [random]*

*1.19 (1.02, 1.40)*

*relative risk (95% confidence interval)*

Cochran Q: P=0.002

I²: 68.2 percent

Egger: P=0.055
