# Supplementary material for: Systematic review: comparative effectiveness of adjunctive devices in patients with ST-segment elevation myocardial infarction undergoing percutaneous coronary intervention of native vessels
Source: BMC Cardiovasc Disord. 2011 Dec 20;11:74. doi: 10.1186/1471-2261-11-74 (PMC3313863; doi:10.1186/1471-2261-11-74)
Supplement: Additional file 36 — Impact of catheter aspiration devices versus control on TIMI-3 blood flow in patients with ST-segment elevation myocardial infarction. Figure of the Impact of catheter aspiration devices versus control on TIMI-3 blood flow in patients with ST-segment elevation myocardial infarction. The squares represent individual point estimates. The size of the square represents the weight given to each study in the meta-analysis. Horizontal lines through each square represent 95 percent confidence intervals. The diamond represents the combined results. The solid vertical line extending from 1 is the null value. [file 1471-2261-11-74-S36.DOC]

*0.2*

*0.5*

*1*

*2*

*Dudek, 2004*

*0.97 (0.79, 1.21)*

*Noel, 2005*

*1.19 (0.96, 1.55)*

*Burzotta, 2005*

*1.18 (0.94, 1.51)*

*Silva-Orrego, 2006*

*1.14 (0.99, 1.33)*

*Kaltoft, 2006*

*1.02 (0.92, 1.14)*

*De Luca, 2006*

*1.15 (0.88, 1.55)*

*Svilaas, 2008*

*1.04 (0.99, 1.10)*

*Ikari, 2008*

*1.09 (0.99, 1.20)*

*Chevalier, 2008*

*1.06 (0.93, 1.21)*

*Sardella, 2009*

*1.19 (1.00, 1.42)*

*Lipiecki, 2009*

*0.66 (0.40, 0.98)*

*Liistro, 2009*

*1.17 (1.04, 1.38)*

*Dudek, 2010*

*1.08 (0.96, 1.23)*

*combined [random]*

*1.08 (1.04, 1.12)*

*relative risk (95% confidence interval)*

Cochran Q: P=0.329

I²: 11.5 percent

Egger: P=0.585
