# Supplementary material for: Systematic review: comparative effectiveness of adjunctive devices in patients with ST-segment elevation myocardial infarction undergoing percutaneous coronary intervention of native vessels
Source: BMC Cardiovasc Disord. 2011 Dec 20;11:74. doi: 10.1186/1471-2261-11-74 (PMC3313863; doi:10.1186/1471-2261-11-74)
Supplement: Additional file 37 — Impact of mechanical thrombectomy devices versus control on TIMI- 3 blood flow in patients with ST-segment elevation myocardial infarction. Figure of the Impact of mechanical thrombectomy devices versus control on TIMI- 3 blood flow in patients with ST-segment elevation myocardial infarction. The squares represent individual point estimates. The size of the square represents the weight given to each study in the meta-analysis. Horizontal lines through each square represent 95 percent confidence intervals. The diamond represents the combined results. The solid vertical line extending from 1 is the null value. [file 1471-2261-11-74-S37.DOC]

*0.5*

*1*

*2*

*Napodano, 2003*

*0.98 (0.86, 1.10)*

*Lefèvre, 2005*

*1.08 (0.99, 1.18)*

*Ali, 2006*

*0.94 (0.89, 0.98)*

*Migliorini, 2010*

*0.94 (0.86, 1.02)*

*combined [random]*

*0.98 (0.92, 1.04)*

*relative risk (95% confidence interval)*

Cochran Q: P=0.026

I²: 67.5 percent

Egger: P=0.464
