# Supplementary material for: Systematic review: comparative effectiveness of adjunctive devices in patients with ST-segment elevation myocardial infarction undergoing percutaneous coronary intervention of native vessels
Source: BMC Cardiovasc Disord. 2011 Dec 20;11:74. doi: 10.1186/1471-2261-11-74 (PMC3313863; doi:10.1186/1471-2261-11-74)
Supplement: Additional file 38 — Impact of distal filter embolic protection devices versus control on TIMI-3 blood flow in patients with ST-segment elevation myocardial infarction. Figure of the Impact of distal filter embolic protection devices versus control on TIMI-3 blood flow in patients with ST-segment elevation myocardial infarction. The squares represent individual point estimates. The size of the square represents the weight given to each study in the meta-analysis. Horizontal lines through each square represent 95 percent confidence intervals. The diamond represents the combined results. The solid vertical line extending from 1 is the null value. [file 1471-2261-11-74-S38.DOC]

*0.5*

*1*

*2*

*Lefevre, 2004*

*0.94 (0.78, 1.11)*

*Guetta, 2007*

*0.94 (0.80, 1.08)*

*Cura, 2007*

*0.92 (0.77, 1.07)*

*Kelbaek, 2008*

*1.11 (1.05, 1.17)*

*Ito, 2010*

*1.17 (0.85, 1.73)*

*combined [random]*

*1.00 (0.90, 1.11)*

*relative risk (95% confidence interval)*

Cochran Q: P=0.011

I²: 69.6 percent

Egger: P=0.252
