# Supplementary material for: Systematic review: comparative effectiveness of adjunctive devices in patients with ST-segment elevation myocardial infarction undergoing percutaneous coronary intervention of native vessels
Source: BMC Cardiovasc Disord. 2011 Dec 20;11:74. doi: 10.1186/1471-2261-11-74 (PMC3313863; doi:10.1186/1471-2261-11-74)
Supplement: Additional file 41 — Impact of catheter aspiration devices versus control on distal embolization in patients with ST-segment elevation myocardial infarction. Figure of the Impact of catheter aspiration devices versus control on distal embolization in patients with ST-segment elevation myocardial infarction. The squares represent individual point estimates. The size of the square represents the weight given to each study in the meta-analysis. Horizontal lines through each square represent 95 percent confidence intervals. The diamond represents the combined results. The solid vertical line extending from 1 is the null value. [file 1471-2261-11-74-S41.DOC]

*0.1*

*0.2*

*0.5*

*1*

*2*

*5*

*10*

*100*

*Burzotta, 2005*

*0.49 (0.16, 1.43)*

*Silva-Orrego, 2006*

*0.29 (0.10, 0.78)*

*Lee, 2006*

*2.96 (0.71, 12.53)*

*Kaltoft, 2006*

*1.51 (0.58, 3.97)*

*Ikari, 2008*

*0.54 (0.36, 0.81)*

*Chevalier, 2008*

*0.54 (0.27, 1.04)*

*Sardella, 2009*

*0.36 (0.24, 0.54)*

*Lipiecki, 2009*

*0.80 (0.17, 3.66)*

*Liistro, 2009*

*0.29 (0.11, 0.78)*

*Dudek, 2010*

*0.98 (0.31, 3.08)*

*combined [random]*

*0.56 (0.39, 0.79)*

*relative risk (95% confidence interval)*

Cochran Q: P=0.069

I²: 43.4 percent

Egger: P=0.161
