# Supplementary material for: Systematic review: comparative effectiveness of adjunctive devices in patients with ST-segment elevation myocardial infarction undergoing percutaneous coronary intervention of native vessels
Source: BMC Cardiovasc Disord. 2011 Dec 20;11:74. doi: 10.1186/1471-2261-11-74 (PMC3313863; doi:10.1186/1471-2261-11-74)
Supplement: Additional file 42 — Impact of mechanical thrombectomy devices versus control on distal embolization in patients with ST-segment elevation myocardial infarction. Figure of the Impact of mechanical thrombectomy devices versus control on distal embolization in patients with ST-segment elevation myocardial infarction. The squares represent individual point estimates. The size of the square represents the weight given to each study in the meta-analysis. Horizontal lines through each square represent 95 percent confidence intervals. The diamond represents the combined results. The solid vertical line extending from 1 is the null value. [file 1471-2261-11-74-S42.DOC]

*0.01*

*0.1*

*0.2*

*0.5*

*1*

*2*

*Napodano, 2003*

*0.29 (0.07, 1.13)*

*Lefèvre, 2005*

*0.21 (0.05, 0.81)*

*Ali, 2006*

*0.84 (0.38, 1.86)*

*combined [random]*

*0.44 (0.17, 1.12)*

*relative risk (95% confidence interval)*

Cochran: P=0.181

I²: 41.6 percent

Egger: Too few strata
