# Supplementary material for: Systematic review: comparative effectiveness of adjunctive devices in patients with ST-segment elevation myocardial infarction undergoing percutaneous coronary intervention of native vessels
Source: BMC Cardiovasc Disord. 2011 Dec 20;11:74. doi: 10.1186/1471-2261-11-74 (PMC3313863; doi:10.1186/1471-2261-11-74)
Supplement: Additional file 43 — Impact of distal balloon embolic protection devices versus control on distal embolization in patients with ST-segment elevation myocardial infarction. Figure of the Impact of distal balloon embolic protection devices versus control on distal embolization in patients with ST-segment elevation myocardial infarction. The squares represent individual point estimates. The size of the square represents the weight given to each study in the meta-analysis. Horizontal lines through each square represent 95 percent confidence intervals. The diamond represents the combined results. The solid vertical line extending from 1 is the null value. [file 1471-2261-11-74-S43.DOC]

*0.1*

*0.2*

*0.5*

*1*

*2*

*5*

*Stone, 2005*

*1.60 (0.85, 3.03)*

*Muramatsu, 2007*

*0.55 (0.18, 1.74)*

*Matsuo, 2007*

*1.16 (0.35, 3.86)*

*Hahn, 2007*

*0.70 (0.24, 1.99)*

*combined [random]*

*1.10 (0.67, 1.81)*

*relative risk (95% confidence interval)*

Cochran Q: P=0.364

I²: 5.8 percent

Egger: P=0.176
