# Supplementary material for: Systematic review: comparative effectiveness of adjunctive devices in patients with ST-segment elevation myocardial infarction undergoing percutaneous coronary intervention of native vessels
Source: BMC Cardiovasc Disord. 2011 Dec 20;11:74. doi: 10.1186/1471-2261-11-74 (PMC3313863; doi:10.1186/1471-2261-11-74)
Supplement: Additional file 45 — Impact of catheter aspiration devices versus control on no reflow in patients with ST-segment elevation myocardial infarction. Figure of the Impact of catheter aspiration devices versus control on no reflow in patients with ST-segment elevation myocardial infarction. The squares represent individual point estimates. The size of the square represents the weight given to each study in the meta-analysis. Horizontal lines through each square represent 95 percent confidence intervals. The diamond represents the combined results. The solid vertical line extending from 1 is the null value. [file 1471-2261-11-74-S45.DOC]

*0.01*

*0.1*

*0.2*

*0.5*

*1*

*2*

*5*

*10*

*Noel, 2005*

*0.31 (0.08, 1.16)*

*Burzotta, 2005*

*0.68 (0.22, 2.11)*

*Silva-Orrego, 2006*

*0.18 (0.05, 0.70)*

*Lee, 2006*

*1.64 (0.45, 6.04)*

*Ikari, 2008*

*0.64 (0.39, 1.05)*

*Chevalier, 2008*

*0.33 (0.12, 0.93)*

*Liistro, 2009*

*0.20 (0.05, 0.78)*

*Dudek, 2010*

*0.58 (0.28, 1.17)*

*combined [random]*

*0.52 (0.35, 0.76)*

*relative risk (95% confidence interval)*

Cochran Q: P=0.307

I²: 15.7 percent

Egger: P=0.278
