# Supplementary material for: Systematic review: comparative effectiveness of adjunctive devices in patients with ST-segment elevation myocardial infarction undergoing percutaneous coronary intervention of native vessels
Source: BMC Cardiovasc Disord. 2011 Dec 20;11:74. doi: 10.1186/1471-2261-11-74 (PMC3313863; doi:10.1186/1471-2261-11-74)
Supplement: Additional file 46 — Impact of mechanical thrombectomy devices versus control on no reflow in patients with ST-segment elevation myocardial infarction. Figure of the Impact of mechanical thrombectomy devices versus control on no reflow in patients with ST-segment elevation myocardial infarction. The squares represent individual point estimates. The size of the square represents the weight given to each study in the meta-analysis. Horizontal lines through each square represent 95 percent confidence intervals. The diamond represents the combined results. The solid vertical line extending from 1 is the null value. [file 1471-2261-11-74-S46.DOC]

*0.01*

*0.1*

*0.2*

*0.5*

*1*

*2*

*5*

*Napodano, 2003*

*0.20 (0.03, 1.23)*

*Lefèvre, 2005*

*0.31 (0.09, 1.00)*

*Ali, 2006*

*1.21 (0.40, 3.67)*

*combined [random]*

*0.50 (0.17, 1.48)*

*relative risk (95% confidence interval)*

Cochran Q: P=0.180

I²: 41.7 percent

Egger: Too few strata
