# Supplementary material for: Systematic review: comparative effectiveness of adjunctive devices in patients with ST-segment elevation myocardial infarction undergoing percutaneous coronary intervention of native vessels
Source: BMC Cardiovasc Disord. 2011 Dec 20;11:74. doi: 10.1186/1471-2261-11-74 (PMC3313863; doi:10.1186/1471-2261-11-74)
Supplement: Additional file 49 — Impact of embolic protection devices combined versus control on no reflow in patients with ST-segment elevation myocardial infarction. Figure of the Impact of embolic protection devices combined versus control on no reflow in patients with ST-segment elevation myocardial infarction. The squares represent individual point estimates. The size of the square represents the weight given to each study in the meta-analysis. Horizontal lines through each square represent 95 percent confidence intervals. The diamond represents the combined results. The solid vertical line extending from 1 is the null value. [file 1471-2261-11-74-S49.DOC]

*0.01*

*0.1*

*0.2*

*0.5*

*1*

*2*

*5*

*10*

*Lefevre, 2004*

*0.29 (0.04, 1.92)*

*Stone, 2005*

*0.17 (0.03, 1.06)*

*Muramatsu, 2007*

*0.32 (0.08, 1.38)*

*Matsuo, 2007*

*1.39 (0.28, 6.82)*

*Hahn, 2007*

*1.05 (0.11, 9.67)*

*Cura, 2007*

*1.00 (0.18, 5.55)*

*combined [random]*

*0.53 (0.24, 1.18)*

*relative risk (95% confidence interval)*

Cochran Q: P=0.603

I²: 0 percent

Egger: P=0.969
