# Supplementary material for: Systematic review: comparative effectiveness of adjunctive devices in patients with ST-segment elevation myocardial infarction undergoing percutaneous coronary intervention of native vessels
Source: BMC Cardiovasc Disord. 2011 Dec 20;11:74. doi: 10.1186/1471-2261-11-74 (PMC3313863; doi:10.1186/1471-2261-11-74)
Supplement: Additional file 50 — Impact of catheter aspiration devices on coronary dissection versus control in patients with ST-segment elevation myocardial infarction. Figure of the Impact of catheter aspiration devices on coronary dissection versus control in patients with ST-segment elevation myocardial infarction. The squares represent individual point estimates. The size of the square represents the weight given to each study in the meta-analysis. Horizontal lines through each square represent 95 percent confidence intervals. The diamond represents the combined results. The solid vertical line extending from 1 is the null value. [file 1471-2261-11-74-S50.DOC]

*0.01*

*0.1*

*0.2*

*0.5*

*1*

*2*

*5*

*Silva-Orrego, 2006*

*0.11 (0.00, 0.94)*

*De Luca, 2006*

*3.00 (0.27, infinity)*

*Svilaas, 2008*

** (excluded)*

*Ikari, 2008*

*0.26 (0.09, 0.72)*

*Chao, 2008*

*0.33 (0.00, 3.76)*

*combined [random]*

*0.30 (0.12, 0.75)*

*relative risk (95% confidence interval)*

Cochran Q: P=0.464

I²: 0 percent

Egger: P=0.626
