# Supplementary material for: Systematic review: comparative effectiveness of adjunctive devices in patients with ST-segment elevation myocardial infarction undergoing percutaneous coronary intervention of native vessels
Source: BMC Cardiovasc Disord. 2011 Dec 20;11:74. doi: 10.1186/1471-2261-11-74 (PMC3313863; doi:10.1186/1471-2261-11-74)
Supplement: Additional file 52 — Impact of catheter aspiration devices on side branch occlusion versus control in patients with ST-segment elevation myocardial infarction. Figure of the Impact of catheter aspiration devices on side branch occlusion versus control in patients with ST-segment elevation myocardial infarction. The squares represent individual point estimates. The size of the square represents the weight given to each study in the meta-analysis. Horizontal lines through each square represent 95 percent confidence intervals. The diamond represents the combined results. The solid vertical line extending from 1 is the null value. [file 1471-2261-11-74-S52.DOC]

*0.1*

*0.2*

*0.5*

*1*

*2*

*5*

*10*

*Svilaas, 2008*

*1.25 (0.37, 4.29)*

*Chevalier, 2008*

*1.08 (0.19, 6.02)*

*combined [random]*

*1.19 (0.40, 3.54)*

*relative risk (95% confidence interval)*

Cochran Q: P=0.898

I²: Too few strata

Egger: Too few strata
