# Supplementary material for: Systematic review: comparative effectiveness of adjunctive devices in patients with ST-segment elevation myocardial infarction undergoing percutaneous coronary intervention of native vessels
Source: BMC Cardiovasc Disord. 2011 Dec 20;11:74. doi: 10.1186/1471-2261-11-74 (PMC3313863; doi:10.1186/1471-2261-11-74)
Supplement: Additional file 54 — Impact of embolic protection devices combined on side branch occlusion versus control in patients with ST-segment elevation myocardial infarction. Figure of the Impact of embolic protection devices combined on side branch occlusion versus control in patients with ST-segment elevation myocardial infarction. The squares represent individual point estimates. The size of the square represents the weight given to each study in the meta-analysis. Horizontal lines through each square represent 95 percent confidence intervals. The diamond represents the combined results. The solid vertical line extending from 1 is the null value. [file 1471-2261-11-74-S54.DOC]

*0.2*

*0.5*

*1*

*2*

*5*

*10*

*100*

*Stone, 2005*

*0.91 (0.60, 1.39)*

*Matsuo, 2007*

*1.85 (0.25, 13.97)*

*Cura, 2007*

*0.33 (0.00, 3.80)*

*combined [random]*

*0.91 (0.60, 1.39)*

*relative risk (95% confidence interval)*

Cochran Q: P=0.697

I²: 0 percent

Egger: Too few strata
